# An Integrative Model for Phytochrome B Mediated Photomorphogenesis: From Protein Dynamics to Physiology

**DOI:** 10.1371/journal.pone.0010721

**Published:** 2010-05-19

**Authors:** Julia Rausenberger, Andrea Hussong, Stefan Kircher, Daniel Kirchenbauer, Jens Timmer, Ferenc Nagy, Eberhard Schäfer, Christian Fleck

**Affiliations:** 1 Centre for Biological Systems Analysis, University of Freiburg, Freiburg, Germany; 2 Institute of Biology II, University of Freiburg, Freiburg, Germany; 3 Institute of Physics, University of Freiburg, Freiburg, Germany; 4 Freiburg Institute for Advanced Studies, University of Freiburg, Freiburg, Germany; 5 Institute of Plant Biology, Biological Research Center, Szeged, Hungary; 6 School of Biological Sciences, University of Edinburgh, Edinburgh, United Kingdom; United States Department of Agriculture-Agricultural Research Service, United States of America

## Abstract

**Background:**

Plants have evolved various sophisticated mechanisms to respond and adapt to changes of abiotic factors in their natural environment. Light is one of the most important abiotic environmental factors and it regulates plant growth and development throughout their entire life cycle. To monitor the intensity and spectral composition of the ambient light environment, plants have evolved multiple photoreceptors, including the red/far-red light-sensing phytochromes.

**Methodology/Principal Findings:**

We have developed an integrative mathematical model that describes how phytochrome B (phyB), an essential receptor in *Arabidopsis thaliana*, controls growth. Our model is based on a multiscale approach and connects the mesoscopic intracellular phyB protein dynamics to the macroscopic growth phenotype. To establish reliable and relevant parameters for the model phyB regulated growth we measured: accumulation and degradation, dark reversion kinetics and the dynamic behavior of different nuclear phyB pools using in vivo spectroscopy, western blotting and Fluorescence Recovery After Photobleaching (FRAP) technique, respectively.

**Conclusions/Significance:**

The newly developed model predicts that the phyB-containing nuclear bodies (NBs) (i) serve as storage sites for phyB and (ii) control prolonged dark reversion kinetics as well as partial reversibility of phyB Pfr in extended darkness. The predictive power of this mathematical model is further validated by the fact that we are able to formalize a basic photobiological observation, namely that in light-grown seedlings hypocotyl length depends on the total amount of phyB. In addition, we demonstrate that our theoretical predictions are in excellent agreement with quantitative data concerning phyB levels and the corresponding hypocotyl lengths. Hence, we conclude that the integrative model suggested in this study captures the main features of phyB-mediated photomorphogenesis in *Arabidopsis*.

## Introduction

One of the major tasks of systems biology is to establish the mechanistic link between mesoscale protein dynamics derived from the genotype and macroscale physiological response of the phenotype. In many experiments in developmental and molecular biology, an organism is manipulated on small scale, e.g., by gene knock-outs or protein mutations. In contrast, however, the corresponding phenotype is observed as a read-out. In order to bridge this gap, a quantitative understanding of this correspondence on multiple spatial as well as temporal scales is necessary. In plants, the phytochrome photoreceptor system epitomizes an excellent model system, where non-invasive manipulation via defined light exposure influences several measurable molecular parameters on the mesoscale, which finally result in macroscopic physiological responses. As sessile organisms tied to the environment, plants have evolved a variety of sophisticated mechanisms to adapt to changes in the local conditions. In particular, the choice of the developmental program of the seedling strongly depends on environmental factors, such as light conditions. Dark-grown seedlings exhibit long hypocotyls, small and closed cotyledons with undifferentiated chloroplasts as well as undifferentiated roots due to repression of light-regulated genes [1]. During photomorphogenesis the activation of light-regulated genes inhibits hypocotyl growth and promotes cotyledon opening and expansion, and chloroplast differentiation. To monitor light quality and quantity, plants have developed several photoreceptor classes: the UV-B photoreceptors [2], the blue/UV-A light absorbing cryptochromes [3], phototropins [4] and the red light absorbing phytochromes [5]. In *Arabidopsis thaliana*, phytochromes are encoded by a small gene family of five members, namely phytochrome A–E [6]. The main photoreceptor mediating photomorphogenesis in red light is phyB [7].

Via photon absorption, the photoreceptor can reversibly switch back and forth between two conformational states. The receptor is synthesized in its red light absorbing form Pr exhibiting an absorption maximum at 660 nm, and forms dimers directly upon synthesis. Under red light exposure, light absorption drives Pr to the far-red absorbing form Pfr, with maximal absorption at 720 nm, which is considered to be the biologically active form [8]. Upon absorption of another photon in far-red light, Pfr is transformed back to the inactive Pr form [9]. Due to the distinct but overlapping absorption spectra of both conformers, the photoequilibrium, i.e., the ratio of Pr to Pfr in steady state is established as defined by the spectral composition of the incident light. Therefore, the intensity, the spectral composition, and the duration of the irradiation represent variable parameters, which can be used to alter the protein dynamics and then to study the emerging effects on the physiological response. However, in addition to the photochemical reactions, phyB-controlled signaling is associated with diverse biochemical reactions. After the onset of irradiation, photoconverted and active phyB Pfr is imported into the nucleus [10], where it forms transient phyB-containing nuclear bodies (phyB-NBs). Fluorescence microscopy revealed that formation of these transient NBs depends on the abundance of basic helix-loop-helix (bHLH) transcription factors, including PIF3 (phytochrome-interacting-factor-3), a negative regulator of photomorphogenesis [11]. Subsequently, a second type, PIF3-independent phyB-NBs are formed, which then remain stable in continuous irradiation of Pfr-promoting wavelengths [11]–[15]. However, the biological role of these phyB-NBs in photomorphogenesis is still under intensive debate [16]. In this study we performed experiments only in continuous irradiation. This condition is not suitable for studying transient NBs, thus here we focus on analyzing properties and functional importance of the stable, later appearing phyB-NBs in regulating phyB signaling.

During the last decade it became clear that improvement of our understanding of phytochrome-controlled signaling requires detailed knowledge about the dynamics of the various pools of different phytochromes. Pioneering studies [17]–[21] provided some information about the functional importance of the dynamics of the light-labile phytochrome A in controlling light-induced signaling; yet despite these efforts our knowledge is still incomplete and quantitative models capturing the main features of phytochrome-mediated signaling are missing. As these statements are also true for phyB-controlled signaling, here we report our efforts aimed at describing, for the first time, phyB-mediated photomorphogenesis in a quantitative way. Our approach took into consideration the following factors. First, at the cellular level the physiological response to irradiation is manifested as the inhibition of cell elongation. However, the molecular events underlying this physiological response are assumed to be controlled by the dynamic interactions of different phyB pools, localized in the nucleoplasm, the nuclear bodies and possibly in the cytoplasm. Second, in the majority of experimental studies, the final hypocotyl length is used as a read-out for phytochrome signaling. To address these issues we developed separate models that describe the macroscopic hypocotyl growth as well as that of the dynamics of phyB protein pools. Construction of these models required obtaining time-resolved data to quantitatively describe these processes. This was achieved by employing various experimental techniques, including FRAP [22] experiments on single nuclear phyB-NBs, immunoblot analysis and in vivo spectroscopy. Finally, the connection between the two separate models, which are on different scales, was achieved by integrating the phyB protein pool dynamics into the growth rate of the hypocotyl growth function.

We note that these models make it feasible to formalize our knowledge about a basic photobiological response, i.e., light-dependent hypocotyl growth inhibition both at physiological and molecular levels. More importantly, the model identifies important components of the molecular network, such as dark reversion rate and abundance of the photoreceptor and allowed us to develop novel interpretations for the function of the stable, persistent NBs. Taken together, we believe that the combined experimental and theoretical approach presented in this study holds the promise to improve our understanding of *how* phyB-mediated photomorphogenesis might work *in vivo*.

## Results

### Kinetic model for phyB protein dynamics

**Figure 1:**
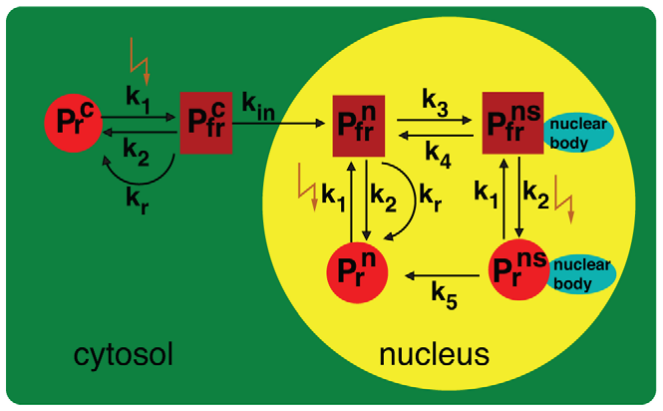
Reaction scheme of phyB dynamics. The superscripts c, n and ns denote the pools of phyB in the cytosol, nucleus and the nuclear bodies, respectively. The inactive and active state of the phyB conformer is denoted by Pr and Pfr, respectively. All transition parameters are described in the text.

Experiments using glucocorticoid receptor phyB fusion proteins demonstrated that the biologically active Pfr conformers located in the cytosol do not trigger physiological responses [23]. In contrast, constitutive nuclear phyB – with an artificial nuclear localization sequence (NLS) – can complement the hypocotyl phenotype of the phyB mutant after light activation [24]. These data indicate that the cytosolic pool of phyB is dispensable for the responses tested in these studies. In addition to the cytosolic phyB pool, under continuous irradiation of Pfr-promoting wavelengths [11]–[15], phyB Pfr localized in the nucleus is associated with NBs but can also be detected in the nucleoplasm. In dark grown seedlings, the Pr form of the phyB-NLS fusion protein is only in the nucleoplasm, which prevents its association to NBs (Kirchenbauer, Kircher, Nagy, Schäfer; unpublished data). Accordingly, six different intracellular pools of phyB can be distinguished after appropriate irradiation, namely cytosolic, diffusely distributed Pr and Pfr conformers, named Pr^c^ and Pfr^c^, diffusely distributed phyB Pr and Pfr localized in the nucleoplasm, i.e., Pr^n^ and Pfr^n^, and NB-bound phyB Pr and Pfr, i.e., Pr^ns^ and Pfr^ns^, cf. reaction scheme of Figure 1.

To keep the protein pool model simple, we neglected dimerization of phytochromes [17], [18], which is known to be rapid. Because diffusion of the phytochromes is fast relative to the dynamics under consideration [25], it is sufficient to describe phytochrome dynamics by the following ordinary differential equations (ODEs) using mass action kinetics:
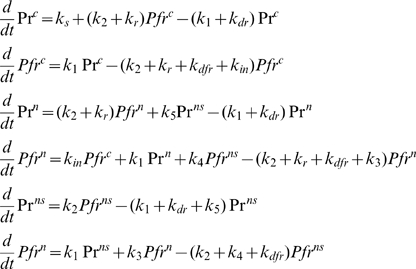
(1)Cytosolic phytochrome is synthesized in its inactive Pr^c^ form at rate k_s_. Under exposure to red light Pr^c^ undergoes a conformational change to Pfr^c^ with transition rate k_1_, depending on light intensity and spectral composition. Subsequently, Pfr^c^ is imported into the nucleus at rate k_in_, where it represents the diffuse form Pfr^n^. In the nucleus, the diffuse form Pfr^n^ can either be reverted back to Pr^n^ at dark reversion rate k_r_ or can accumulate at rate k_3_ into nuclear bodies Pfr^ns^, which decompose at rate k_4_. Due to the overlapping absorption spectra of Pr and Pfr, Pfr^n^ and Pfr^ns^ are partially reverted at the light-dependent transition rate k_2_ to Pr^n^ and Pr^ns^, respectively, where the latter decomposes to Pr^n^ at rate k_5_. The association and dissociation as well as the dark reversion of Pfr to Pr occur independently of light and therefore become important in darkness and weak irradiation. All Pr and Pfr pools are linearly degraded at rates k_dr_ and k_dfr_, respectively. The light intensity and wavelength dependent rate constants k_1_ and k_2_ are given by k_1_ = N_λ_σ_r_ and k_2_ = N_λ_σ_fr_, where N_λ_ describes the photon flux at wavelength λ and σ_r/fr_ denote the photoconversion cross-sections [19], [26].

### Experimental characterization of protein dynamics and hypocotyl growth

In order to obtain time-resolved resolution of phyB dynamic behavior we performed experiments to address the following questions: (1) Are the diffuse and NB Pfr pools in equilibrium with each other? (2) Can the phyB-containing nuclear bodies be depleted? (3) What is the role of light-dependent phyB degradation? (4) How are these processes linked to the dark reversion mechanism of Pfr inactivation?

#### FRAP experiments on single nuclear bodies show that nuclear pools are in equilibrium

**Figure 2:**
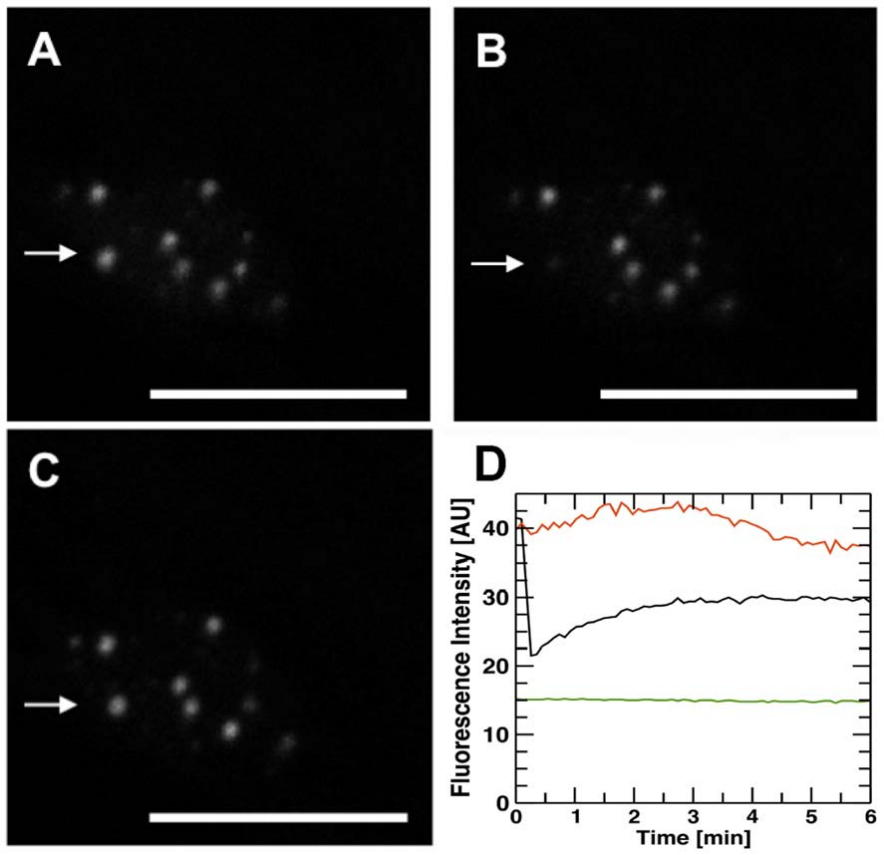
Dynamics of phyB nuclear body formation in *Arabidopsis* seedlings via FRAP analysis. Selected imaging data of a representative FRAP experiment on nuclear bodies of phyB-YFP are shown. Etiolated seedlings were irradiated for 24h with red light prior to analysis in a confocal laser scanning microscope. Nuclear body distribution of the photoreceptor is shown directly before (A), immediately after (B), and 3 min after (C) photobleaching the NB marked by an arrow (scale bar = 10 µm). (D) Representative raw data of FRAP experiments showing the fluorescence intensity of the bleached NB (black), of a non-bleached reference NB (red), and of the background (green) over time.

The dynamics of the diffuse, nuclear phyB Pfr^n^ and the nuclear body-bound, Pfr^ns^ was analyzed by FRAP [22]. Photobleaching the YFP (Yellow Fluorescent Protein) portion of the phyB-YFP fusion protein in precisely defined nuclear areas made it possible to follow potential exchange rates of the Pfr^n^ and Pfr^ns^ pools by confocal laser microscopy. We found that bleaching of Pfr^n^ in the nucleoplasm led to a qualitative loss of Pfr^ns^ fluorescence of NBs in the same nucleus, indicating that Pfr^ns^ can dissociate from stable NBs. Bleaching of a single phyB-NB (Pfr^ns^) also showed a loss of fluorescence of the unbleached NBs, see Figure 2D. Furthermore, the fluorescence of the single, bleached phyB-NB showed almost complete recovery after a few minutes, Figure 2A–D. Therefore, we conclude that diffuse phyB Pfr molecules associate and dissociate from NBs quite rapidly. In addition we note that the loss of intensity of a non-bleached NB in the vicinity of a bleached one indicates that all NBs are in equilibrium with each other via the nucleoplasmic diffuse Pfr pool. For a detailed discussion of the determination of the Pfr exchange rates see File S1.

#### Nuclear body depletion is strongly dependent on the phytochrome conformational state

Recently, it has been reported that both phyB Pr and phyB Pfr containing NBs can be depleted in tobacco [27]. Here we confirmed this finding for *A. thaliana* in a transgenic line expressing the PHYB:GFP green fluorescent fusion protein. In the present study, plants were either irradiated to induce NB formation, followed by an immediate transfer to darkness or, alternatively, after the inductive light treatment, plants were irradiated with a Pr-creating long wavelength far-red light pulse (RG9 pulse) and then transferred to darkness. The effect of these light treatments on the depletion of phyB Pr and Pfr containing NBs was determined by counting the average number of detectable NBs. Our data demonstrate that NBs containing predominantly Pfr were visible over at least 9 h of prolonged darkness, whereas phyB Pr associated NBs were already depleted after 1 h in darkness, see File S1. The differences in the depletion kinetics clearly showed a higher potential of the phyB Pfr form to stay in the NB-bound pool.

**Figure 3:**
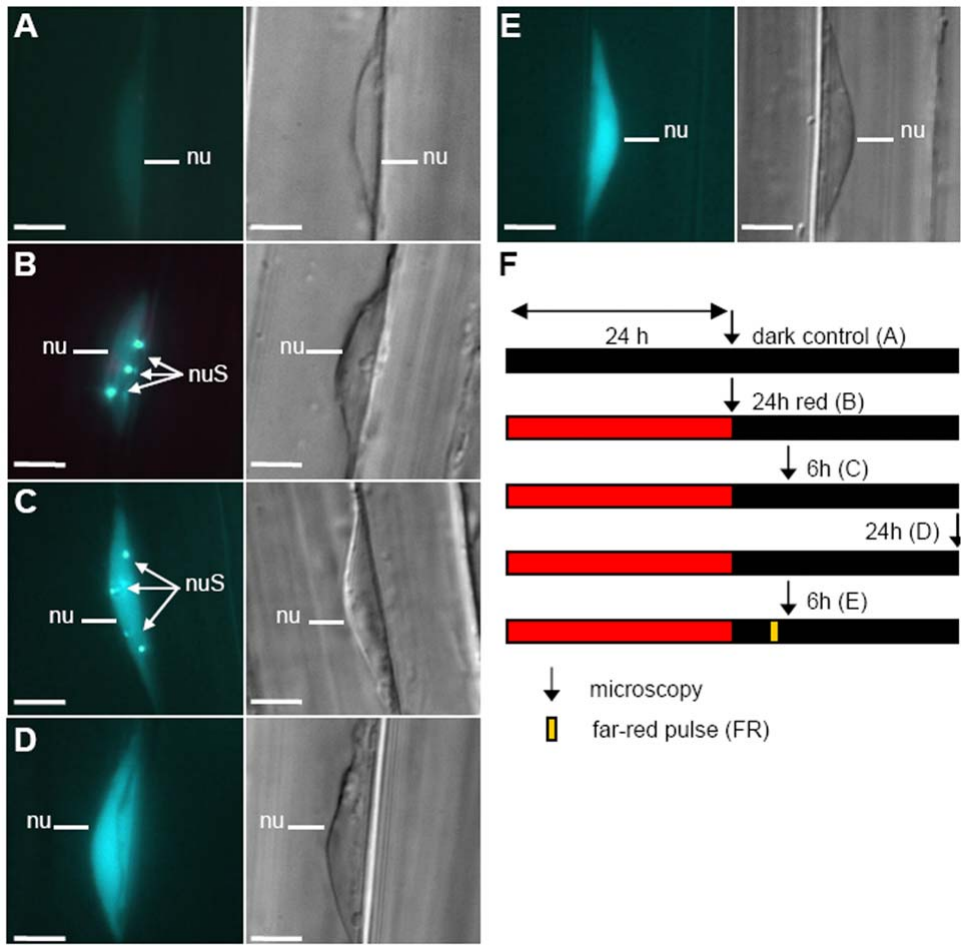
Dynamics of phyB NB formation in *Arabidopsis* seedlings. (A–E) Representative fluorescence microscopy pictures and corresponding DIC images of phyB-GFP expressing transgenic lines show photoreceptor localization of (A) dark grown and (B–E) red light irradiated seedlings (24 h, 30 µmol m^−2^s^−1^). After red light irradiation, seedlings were either transferred to darkness for 6 h (C) or 24 h (D), respectively. Localization after red light irradiation, followed first by 5h darkness, then by a far-red light pulse (5 min, 20 µmol m^−2^s^−1^) and one subsequent hour of darkness is shown in (E). Irradiation with far-red light served to convert Pfr to Pr (nu = nucleus, nuS = nuclear body; scale bar = 10 µm). A schematic representation of the light treatment is shown in (F).

Figure 3A–E shows fluorescence microscopic pictures of phyB localization in dark-grown seedlings (Figure 3A) or after NB-inducing red light irradiation (Figure 3B–E). Figure 3B shows the localization directly after red light treatment, whereas Figs. 3C and D show the localization in darkness, 6h or 24h after the red light treatment. These data demonstrate that NBs are still visible after 6h but not after 24h dark incubation. Furthermore, comparing Figure 3C with Figure 3E, both taken after the same dark period length, reveals that the red light induced NBs contain predominantly phyB-Pfr, because even after 5h darkness, a far-red light pulse promoting formation of phyB Pr (Figure 3E) can lead to a complete loss of detectable fluorescent NBs within an additional hour of darkness.

#### Light-induced Pfr degradation as an additional switch-off mechanism

**Figure 4:**
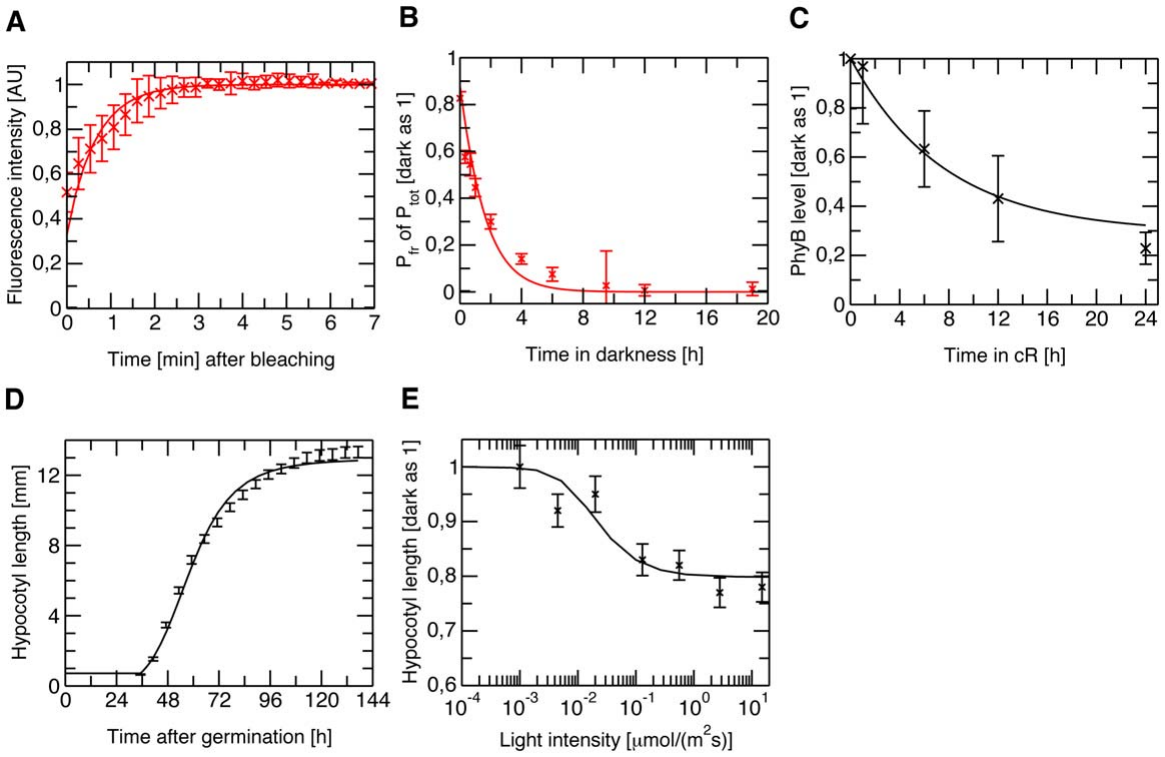
Multi-experiment fit. Experimental data (crosses with error-bars) and the corresponding multi-experiment fit (solid lines) of the phytochrome B dynamics of Col WT (black) and over-expressing lines (red). Error-bars indicate standard errors (SE), based on biological replicates. (A) Time course of FRAP experiments, see Figure 2. (B) Time course of dark reversion kinetics after a saturating red light pulse (5 min, 22 µmol m^−2^s^−1^). Relative Pfr amount in ABO/A^−^ line was measured in a dual-wavelength ratio spectrophotometer [52]. (C) Red light induced phyB degradation. Semi-quantitative quantification of phyB specific immunoblot signals of Col WT seedlings irradiated with continuous red light (3 µmol m^−2^s^−1^) and normalized to dark value is shown. One exemplary blot is shown in Figure S1A. (D) Dark growth curve of seedlings. Single seedlings (Col WT, *phyB-9* mutant, phyB-GFP-1) were imaged over six days in continuous darkness and the mean hypocotyl length is presented. (E) Fluence rate response curve. Relative hypocotyl length, normalized to dark-grown seedlings, of Col WT upon growth in different fluence rates of continuous red light for four days is shown. For all experiments, we established appropriate models, presented in File S1, and performed a simultaneous multi-experiment fit to the experimental data.

In order to further characterize the phyB kinetics we monitored the light-induced degradation of phyB after exposing dark grown seedlings to continuous red (cR) light. The data obtained by immunoblot analysis are shown in Figure 4C and Figure S1A. These figures demonstrate that phyB level decreases by 60% within 24 h of red light treatment as compared to etiolated seedlings, which is consistent with results published by Khanna et al. [28], Leivar and colleagues [29] or Sharrock and Clark [30]. These observations suggest that for understanding phyB-driven physiology, the degradation kinetics of the photoreceptor has to be taken into account. We note that light-dependent phyB degradation can be interpreted as an additional Pfr inactivation mechanism, comparable to phytochrome A, where this mechanism has become more prominent [18].

#### *In vivo* dark reversion is much slower than *in vitro*

Kunkel et al. [31] and Sweere et al. [32] measured the light-independent reversion of *A. thaliana* phyB Pfr to Pr (so-called dark reversion) and reported a relatively rapid initial dark reversion within the first 20 minutes of darkness in yeast cells. In addition, Sweere and colleagues [32] found an augmented half-life time of about 60 min, when dark reversion experiments were performed *in vivo* in etiolated *A. thaliana* seedlings. In this study, we analyzed the reversion of phyB Pfr in prolonged darkness for up to 24 h after a strong red light pulse in etiolated seedlings (5 min, 22 µmol m^−2^s^−1^). Due to spectroscopically interfering chlorophyll formation under prolonged irradiation it was not possible to measure dark reversion after light treatments inducing formation of NBs. We show, however, that under non-NB-inducing light conditions most of the Pfr reverts in the first 4 h to 6 h (data of Figure 4B) leading to an overall half-life of about 60 min. This finding is in excellent agreement with the experimental data reported by Sweere et al. [32], but is not consistent with a single first order reaction with a half-life of 20 min as described for *A. thaliana* phyB expressed in yeast [31]. The prolonged persistence of phyB in its Pfr form, as described above, suggests the action of a stabilization mechanism, which protects NB-bound phyB Pfr from dark reversion *in planta*. This hypothesis is additionally supported by the observation that after a dark period of 5h, a single far-red light pulse leads to the disappearance of the NBs induced by a previous red light treatment (see Figure 3E).

#### Experiments on time-resolved hypocotyl growth

Hypocotyl growth is a standard read-out for phytochrome signaling and it is used to study the fluence rate dependency of hypocotyl elongation at a specific wavelength.

Time-resolved experiments on seedlings grown under light/dark cycles demonstrated that hypocotyl elongation is rhythmic, it follows a diurnal pattern and it is regulated by the circadian clock [33]–[35]. In contrast to light/dark grown seedlings, hypocotyl growth displays no rhythmicity in seedlings irradiated with continuous light or grown in darkness. Thus to avoid optimizing additional parameters we used seedlings imbibed and grown in constant darkness or continuous light. Under these conditions the endogenous circadian clock is not entrained, consequently no rhythmic hypocotyl growth can be observed, Figure 4D and File S1. To support the modeling efforts we recorded the hypocotyl lengths of *A. thaliana* (ecotype Columbia) wild type (Col WT), *phyB-9* mutant and phyB-GFP-1 over-expressor lines in a time-resolved manner (data of Figure 4D shows mean of 13 single seedlings). When grown in darkness, all three genotypes showed the same S-shaped growth pattern (File S1), showing that different phyB levels, present in the inactive Pr form, do not have any influence on growth in darkness. In contrast, final hypocotyl lengths varied, when seedlings were grown under continuous irradiation with red light, see File S1.

### Modeling time-resolved hypocotyl growth

Differences in the phyB signaling network of Eqn. (1) lead to differences in the physiological response. To capture these changes of the hypocotyl growth behavior, we developed a model describing hypocotyl growth. Based on the theoretical ideas of Backman [36] as well as Hock and Mohr [37] about asymmetric organ growth, which is discussed in more detail in File S1, we propose a hypocotyl growth function describing the time evolution of dark-grown seedlings by:
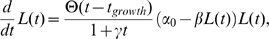
(2)where *L(t)* is the length of the seedling at time *t*. The parameters can be interpreted in the following way: in the absence of any environmental limitations, i.e., β = 0, hypocotyl growth would be exponential with growth rate α_0_(1+γt)^−1^. If β≠0, the initial growth rate is given by α_0_−βL_0_, with the initial hypocotyl length L_0_ = L(t_0_). For increasing hypocotyl length and later times, the environmental limitations become stronger, i.e., growth saturates to the steady state level L(t→∞) = α_0_β^−1^. We also introduced the Heaviside function Θ(t) (Θ(t) = 1 for t>0 and Θ(t) = 0 otherwise), which describes a delayed growth start after germination induction. The fraction α_0_γ^−1^ determines the initial growth profile, see File S1. For α_0_ = γ, the hypocotyl exhibits linear growth, for α_0_>γ it grows exponentially, whereas for α_0_<γ it grows sublinearly. The main effect of γ is to slow down growth for t>γ^−1^ resulting in a flattening of the growth curve for prolonged times. This reflects the experimental observation that the older the organism, the slower the growth towards its final length. The solution of Eqn. (2) is given by
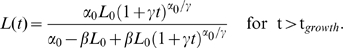
(3)Seedlings grown in darkness as well as in light should be described with one and the same growth function, i.e. one has to incorporate the inhibitory effect of phytochrome into Eqn. (2). A judicious choice is to modify the growth rate of Eqn. (2) to α(t) = α_0_(1+K′^2^ũ^2^(t))^−1^, where ũ(t) represents the active phytochrome and K′^−1^ is the value where the growth rate α(t) has half of its maximal value (for discussion of α(t) see File S1). In the absence of active phytochrome, α(t) reduces to the original α_0_, cf. Eqn. (2). Hence, the time evolution of hypocotyl growth of light-grown seedlings can be described by
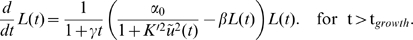
(4)Detailed discussions of the growth model and the explicit solution of Eqn. (4) are given in File S1.

### Parameter estimation and sensitivity analysis

To obtain the quantitative values of the kinetic parameters describing the pool dynamics of Eqn. (1) and the hypocotyl growth given by Eqn. (4), we performed simultaneous nonlinear least-square fitting of the protein and hypocotyl growth model to all experimental data sets (see Materials and Methods). Using model selection methods [38], [39], we exclude the cytosolic Pfr^c^ from relevance for the physiological response. Due to the rapid association and dissociation kinetics of the nuclear Pfr pools (Figure 4A), both nuclear Pfr pools have the potential to trigger downstream signaling and are not distinguishable on the basis of experiments presented here, see File S1. The biologically reasonable parameter ranges, the estimated mean and the 95%-confidence intervals are summarized in Table 1. Figure 4 shows that the independent experiments (crosses with error bars) can be well described simultaneously by assuming that downstream signaling is triggered by nuclear Pfr (solid lines represent the data fit).

**Table 1:** Simultaneously estimated kinetic parameters. Kinetic parameters and photoreceptor abundance P_0_ were either estimated on phytochrome accumulation data presented in File S1 or simultaneously estimated on the basis of the experimental data of Figure 4 and nuclear Pfr to be the signaling component. The last column gives the mean and the 95%-confidence intervals (CI) of the estimated parameters [55].

Parameter [unit]	Biologically reasonable parameter range	Estimated mean (95% CI)
k_dr_ [min^−1^]	-	0.00061 (±0.00032)
P_0_^phyB-GFP−1^ [1]	-	3.8*P_0_^Col^ (−)
k_3_ [min^−1^]	0.1–10	3.98 (±0.028)
k_4_ [min^−1^]	0.1–10	1.51 (±0.203)
k_r_ [min^−1^]	0.001–0.1	0.0321 (±0.0045)
k_dfr_ [min^−1^]	k_dr_–5*k_dr_	0.0024 (±0.0004)
α_0_β^−1^ [mm]	1–15	8.93 (±0.09)
γ [min^−1^]	1–200	141.95 (±3.99)
K [1]	0.1–10	6.87 (±3.36)
L_0_ [mm]	0–1	0.717 (±0.034)
α_0_γ^−1^ [1]		5.66

**Figure 5:**
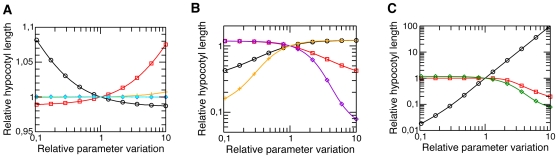
Predicted effect of relative parameter variation on relative hypocotyl length. The parameters were changed from 10% to 1000% of their estimated value. The relative hypocotyl length was determined according to Eqn. (4) and is plotted on the linear scale in (A) and on the logarithmic scale in (B) and (C). (A) Dynamical protein parameters k_1_ (black circle), k_2_ (red square), k_5_ (violet diamond), k_r_ (orange plus), and k_in_ (cyan star), whose relative variation have little effect on the physiological read out. (B) Variation of k_3_ (black circle) or k_4_ (red square), over-expression strength z of the photoreceptor abundance relative to P_0_^Col^ (violet diamond), and Pfr-degradation rate k_dfr_ (orange plus) had stronger effect on the relative hypocotyl length. (C) Variations of the direct growth parameter α_0_β^−1^ (black circle), K (green diamond), and the over-expression strength z (red square).

On the basis of the fitted model, we performed sensitivity analysis to study which parameters of the protein dynamics and the growth model are most relevant for the control of the amount of nuclear Pfr and the final hypocotyl length (normalized to the hypocotyl length for the estimated parameters of Table 1). Figure 5A shows the parameters of the protein dynamics whose variation has little or no effect on relative hypocotyl length, namely k_1_, k_2_, k_5_, k_r_, and k_in_. Furthermore, nuclear phyB Pfr NB formation via k_3_ and dissociation via k_4_ have a strong antagonistic effect on the physiological readout, Figure 5B. Varying the expression strength z = P_0_^Mutant^/P_0_^Col^, where P_0_^Col^ is the wild type and P_0_^Mutant^ the mutant photoreceptor abundance, respectively, has the strongest effect on the relative hypocotyl length. Variation of the Pfr degradation rate k_dfr_ confirms the intuitively expected antagonistic effect on the variation of photoreceptor abundance, Figure 5B. These results show that, if we assume for example diffuse Pfr^n^ as signaling component, a reduced NB association rate k_3_ (Figure 5B, black circle) results in an enhanced amount of Pfr^n^, which in turn leads to a stronger hypocotyl growth inhibition, whereas a weaker hypocotyl growth inhibition is obtained for a higher NB association rate. The contrary holds for the NB dissociation rate k_4_ (Figure 5B, red square). Moreover, if the photoreceptor abundance is lower than the wild-type abundance P_0_^Col^ (Figure 5B, violet diamond), hypocotyl growth inhibition is weaker, whereas a strong over-expression leads to an enhanced hypocotyl growth inhibition. Decreasing the Pfr degradation k_dfr_ (Figure 5B, orange plus) results in an elevated amount of Pfr^n^, which triggers a stronger hypocotyl growth inhibition, whereas an increased k_dfr_ reduces the amount of Pfr^n^, and therefore leads to weaker hypocotyl growth inhibition. Furthermore, we performed sensitivity analysis for the parameters of the hypocotyl growth model, Figure 5C. Since α_0_β^−1^ determines the final hypocotyl length, its variation showed a very strong effect on relative hypocotyl length. An increase of γ of more than 100% led to a decrease of the final hypocotyl length, which is explained by the fact that an increased γ makes hypocotyl growth less steep such that the final hypocotyl length α_0_β^−1^ was not reached during the course of the simulation. Variation of K′ had the same effect as changing the over-expression strength z of the photoreceptor abundance, because all rescaled Pfr components and their sums depend on z, such that the product Kz is decisive for hypocotyl growth.

In the following, we use our proposed mathematical model together with the estimated parameter values to point out two further aspects of the phytochrome B system.

### A Pfr switch-off mechanism is necessary to reproduce the characteristic fluence rate dependent hypocotyl growth inhibition

**Figure 6:**
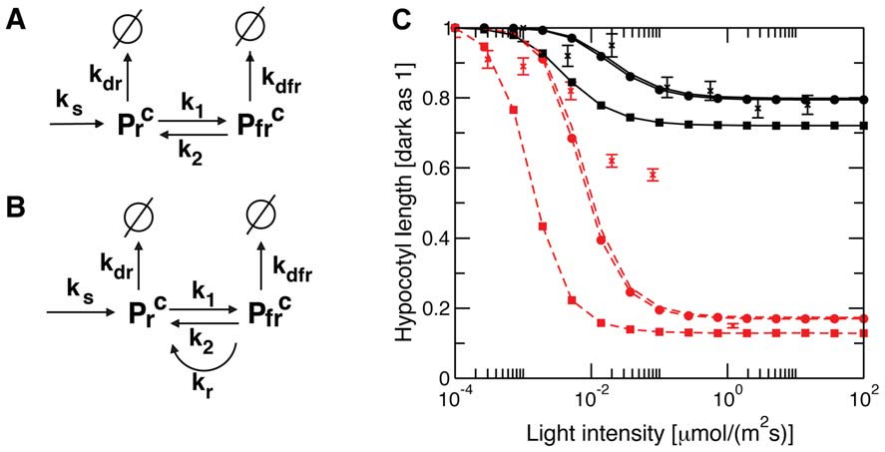
Fluence rate dependency. Submodels of phyB dynamics, describing only the photochemistry (A) or including the additional process of dark reversion (B). (C) The fluence rate response curves for Col WT (black solid lines) were estimated in a multi-experiment fit, whereas the fluence rate response curves of phyB-GFP-1 (red dashed lines) were predicted on the basis of the estimated parameters for different models of phyB dynamics, describing only photochemistry (A, square), including photochemistry and dark reversion kinetics (B, circle), or including the full protein dynamics of Figure 1 (no symbols). Error bars indicate standard error of biological replicates.

Physiological experiments showed that phytochromes not only detect light quality but also operate as light quantity sensors [40]. Furthermore, phyB over-expressing lines exhibit a rather strong hypocotyl growth inhibition at high fluences [40], [41], but still show a similar range of intensity dependence as the wild type (Figure 6C). This finding indicates that the total amount of phyB is limiting for maximal signal strength, but has only a minor effect on the intensity dependency. Therefore, the amount of active signaling Pfr in photoequilibrium cannot mediate the observed light dependency, suggesting at least one additional regulatory step. To find the minimal requirements for a characteristic fluence rate dependent hypocotyl growth inhibition, we analyzed different phyB submodels (Figs. 6A, B), performed a multi-experiment fit for the wild-type fluence rate response curve (Figure 6C, black solid lines), and predicted the fluence rate response curves for the over-expressor phyB-GFP-1 line (Figure 6C, red dashed lines). In the simplest pool model of phytochrome dynamic, phytochrome is synthesized, photoconverted and degraded (Figure 6A) and Pfr represents the signaling component. Considering the photochemistry alone, the hypocotyl growth as described by Eqn. (4) is already inhibited at very low light intensities, contradicting the experimental findings. The simplest extension of the photochemistry model is to assume inactivation of Pfr via dark reversion (Figure 6B). By again considering Pfr as the active signaling component, the estimated wild type and the predicted over-expressor sensitivities of hypocotyl growth inhibition fit the experimentally observed range by showing little inhibition at very low fluences (Figure 6C, circles). Note that the difference between the final hypocotyl lengths at high fluences is due to the fact that we simulated the hypocotyl growth for four-day-old seedlings, which may not have reached their final hypocotyl lengths. In contrast to high fluences the impact of the dark reversion rate k_r_ cannot be neglected for low and intermediate light intensities, see File S1.

Using the full protein dynamics of Eqn. (1) and the corresponding estimated kinetic parameters (Table 1), we again predicted qualitatively correct the fluence rate response behavior for the over-expressor phyB-GFP-1 line (Figure 6C, red-dashed line without symbols). As in the case for the dark reversion submodel (Figure 6B), the experimental inhibition profile of the over-expressing line phyB-GFP-1 is mimicked appropriately.

Therefore, the mathematical analysis of the submodels shows that an additional inactivation step of the active phyB pool, either by dark reversion and/or by NB formation, is required in order to reproduce the experimentally observed sensitivity range of hypocotyl growth inhibition. These results give for the first time a good alternative to the former hypothesis that a phytochrome intermediate formed during photoconversion is the physiologically active phytochrome form [42].

### Nuclear bodies are necessary for slow dark reversion observed in planta

**Figure 7:**
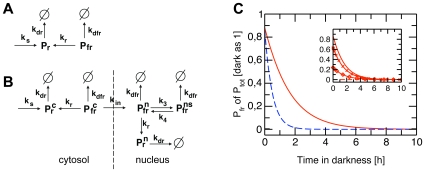
Dark reversion experiments in vitro and in planta. Reaction scheme (A) assuming a single first order reaction or (B) assuming the entire biochemical pool dynamics of the integrative model depicted in Figure 1. (C) Dark reversion curves calculated with the estimated dark reversion rate k_r_ = 0.03 [min^−1^] (see Table 1), for assuming a single first order reaction (blue dashed line) or for the entire model (red solid line). The inset shows the fraction of the single Pfr pools relative to Ptot for (B): Pfr^c^ (dashed line), Pfr^n^ (red diamonds), Pfr^ns^ (red crosses), sum of all (solid line).

As discussed above the experimental findings for the dark reversion of phyB expressed in yeast [31], [32] contradict the observation of prolonged dark reversion in etiolated seedlings (experimental data of Figure 4B). Through our integrative model we are able to offer a reasonable explanation to harmonize these apparently conflicting observations and conclude that complex interdependencies at molecular level mean that dark reversion experiments cannot be described by a simple exponential decay function (Figure 7B). Furthermore, it should be noted that in the dark reversion experiments the sum of all Pfr pools relative to the overall phytochrome abundance is measured. Therefore, the estimation of the dark reversion rate depends on all other parameters of the dynamical phyB system and can only be determined by a multi-experiment fit of several experiments. With this approach we estimated the mean kinetic dark reversion rate to be k_r_ = 0.03 [min^−1^] (see Table 1). In the case of the dark reversion experiment done by Kunkel et al. [31], where phyB was expressed in yeast cells, the assumption to describe this experiment by means of an exponential decay of Pfr to Pr is justified. In this experimental system no additional molecular interactions such as NB formation occur. This permits a direct conversion of the kinetic rate to half-life time and vice versa (Figure 7A). If one uses one and the same estimated k_r_ = 0.03 [min^−1^] of the phyB model presented here, the calculated half-life time in the case of exponential decay is 23 min. Therefore, the kinetic dark reversion rate, which was estimated in etiolated seedlings (Figure 7C, red solid line) is in very good agreement with the rate of dark reversion obtained for phyB expressed in yeast cells (Figure 7C, blue dashed line). Furthermore, the inset of Figure 7C shows that most of the nuclear Pfr is stored in the nuclear bodies (red crosses). Therefore, the theoretical analysis of the model supports the experimentally driven hypothesis that nuclear bodies stabilize Pfr and may serve to protect phyB Pfr from dark reversion.

### The role of photoreceptor abundance in hypocotyl growth experiments

**Figure 8:**
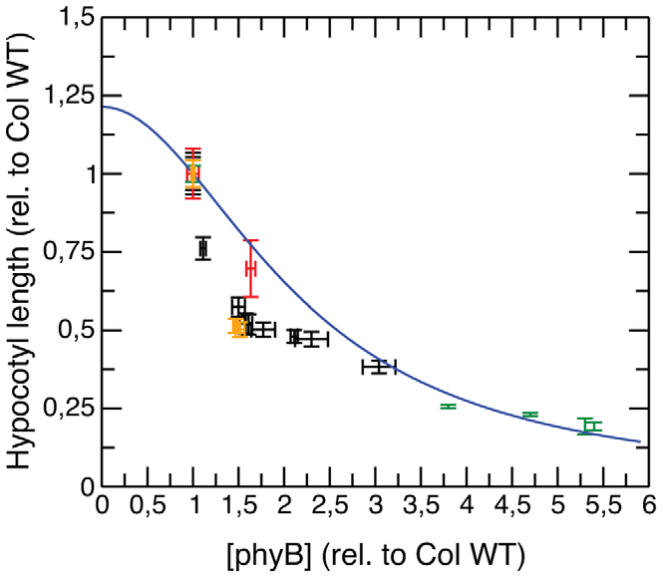
Role of photoreceptor abundance on hypocotyl growth. The experimental data, i.e., phyB levels and hypocotyl lengths, are taken from Khanna et al. (red), Leivar et al. (black), Al-Sady et al. (orange) [28], [29], [46], and were generated in the present work (transgenic over-expressing phyB-GFP-1 to 4 lines, green). The data were normalized to the phyB level and hypocotyl length of Col WT. Using the estimated parameters from Table 1, the simulated prediction (blue solid line) was generated according to Eqn. (6). The mean values and the standard errors (error-bars) are summarized in Table S1.

Over-expression of phyB did not lead to an altered dark phenotype, but resulted in hypersensitivity to irradiation [43]. Recent research has identified potential interaction partners of the phytochromes and partly unraveled a complex phytochrome-controlled signal transduction network [44]. One group of interaction partners consists of the phytochrome interacting factors (PIFs). They are members of the bHLH family and play a crucial role in the phytochrome signal transduction networks [45], although the mechanisms by which the PIFs regulate phytochrome signaling are not yet fully understood. Recent publications, however, suggest that one of their functions is to directly regulate photoreceptor abundance, i.e., the total amount of phytochrome: Khanna et al. [28] reported that *pif5*-mutants contained 1.6-fold higher levels of phyB compared to Col WT. In addition to PIF5, Leivar and colleagues [29] newly identified PIF7, together with PIF3 and PIF4, as well as Al-Sady et al. [46] identified PIF3 to regulate hypocotyl length by modulating phyB levels. The differences in phyB level correlated with the physiological differences in hypocotyl growth inhibition. An increase in phyB level, relative to Col WT, in the single and double *pif* mutants resulted in a strongly inhibited hypocotyl (see Figure 8 and Table S1).

The analysis of our model also indicates that phyB abundance is a key parameter for hypocotyl growth inhibition in continuous irradiation with monochromatic red light (Figure 5B). In order to reveal the functional relationship between photoreceptor abundance and the hypocotyl lengths measured, we performed additional mathematical analysis of the model.

Due to the experimental observation that phytochrome synthesis can be observed approx. 12 h after germination induction (estimated t*_syn_* = 648 min, see File S1), whereas the hypocotyl starts to grow approximately 35 h after germination induction (t*_growth_* = 2111 min, see File S1), the protein dynamics has already reached its steady state level when the hypocotyl starts to grow. Therefore, the solution of the rescaled signaling component u(t) = ũ(t)/P_0_^Col^ becomes time-independent with ū as the steady state level, and the modified growth reads α′ = α_0_(1 + K^2^ū^2^)^−1^, with K = K′ P_0_^Col^. All rescaled steady state concentrations depend linearly on the expression strength z = P_0_^Mutant^/P_0_^Col^ of the photoreceptor, i.e. ū = zf(p), where the function f(p) depends on the set of all parameters p except k_s_. In particular, this means that the function f(p) encompasses the experimental parameters like light intensity, light composition, or duration of the irradiation. It follows that for constant experimental settings the function f(p) is a constant, and the difference with respect to hypocotyl length between the over-expressing line and the wild type is completely captured by the expression strength parameter z. The end-point of the hypocotyl length L′ is the solution of Eqn. (4) and describes the equilibrated level of the hypocotyl. It is given by
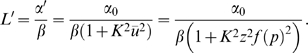
(5)In order to validate the algebraic dependence of relative hypocotyl length on the total amount of phytochrome described by Eqn. (5), we generated transgenic lines (called phyBGFP-1 to 4) expressing the 35S:PHYB:GFP transgene and verified the different expression levels by immunoblot data (see Figure S1B and Table S1). Figure 8 shows the dependence of relative hypocotyl length on the total amount of phytochrome, normalized to Col WT. We combined the *pif*-mutant data of Khanna et al. [28], Al-Sady and colleagues [46] as well as Leivar et al. [29] with our over-expressing lines phyB-GFP-1 to 4 relative to the phyB level of Col WT, since all experiments were conducted under saturating red light conditions for four days.

The hypocotyl lengths of the mutants and transgenic line, L′_M_, were normalized to the Col WT hypocotyl length, L′_Col_, and read in dependence of the over-expression strength z of the photoreceptor abundance relative to P_0_^Col^

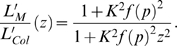
(6)Note that z = 1 for Col WT. Using the estimated parameters obtained from our multi-experiment fit (see Table 1), we calculated the phyB abundance response curve presented in Figure 8 (blue solid line) according to Eqn. (6). We find excellent agreement between the predicted curve and the experimental in-house and published data of over-expression lines in continuous monochromatic red light irradiation.

## Discussion

Despite considerable progress in the identification of signaling chain components and the characterization of their interdependencies by molecular biology approaches, a quantitative understanding of the correspondence between protein dynamics and plant physiology in photomorphogenesis is still missing. The photoreceptor phytochrome B system of *Arabidopsis thaliana* has been intensively investigated during the last decade [8], such that a detailed picture of protein dynamics can be derived leading to an exceptional study system. The combination of experiments and theory presented here provides, for the first time, a quantitative description of the phyB dynamics. Furthermore, we propose a hypocotyl growth function and incorporate the protein dynamics by using a phyB-dependent growth rate. With this approach we are able to span multiple time and length scales necessary for the quantitative analysis of red light induced developmental processes.

An experimentally non-intuitive characteristic of the phytochrome signaling network is the mediation of fluence rate dependency. As the amount of phyB-Pfr is limiting for maximal signal strength, but does not influence the range of intensity dependence, the overall amount of Pfr cannot explain this effect. Our combined approach, however, revealed a theoretical linkage between inactivation of phyB-Pfr, via dark reversion and/or NB formation, and the fluence rate dependent hypocotyl growth inhibition, which is difficult to deduce by experimental results alone.

The experimental observations that (i) the nuclear diffuse and NB-bound Pfr pools are in equilibrium, (ii) the NBs, containing either active or inactive phyB are depleted at different rates indicate that nuclear bodies function as storage sites for phyB Pfr that is released in darkness. This nuclear Pfr storage results (i) in prolonged dark reversion kinetics, which was observed in *A. thaliana* seedlings with a half-life time of about 60 minutes (Figure 4B) as well as (ii) in a partial reversibility of phyB responses in prolonged darkness even after 6 hours, which has already been described [47], [48]. Our integrative model provides, for the first time, a mechanistic explanation for this classical experimental observation. The diffuse cytosolic and nuclear Pfr pools, however, revert relatively fast upon transfer to darkness, which has been shown for *A. thaliana* phyB expressed in yeast cells with a half-life time of about 20 minutes [31]. Therefore, the theoretical analysis helps to elucidate these non-intuitive experimental results and provides an explanation that resolves this apparent discrepancy.

Using the photoreceptor protein model in combination with a model for hypocotyl growth for parameter estimation, we successfully connect protein dynamics to the corresponding macroscopic physiological response. Variation of all biological parameters revealed that the proposed relationship between protein dynamics and hypocotyl growth is most sensitive to direct perturbations in the photoreceptor abundance P_0_. Therefore, our integrative model theoretically formalizes previously known biological knowledge on the relationship between photoreceptor abundance and hypocotyl length, Eqn. (6). Its prediction is in excellent agreement with experimental in-house and published over-expression data (Figure 8), which can be considered as a test of the model's robustness. This implies that our model captures the essential features of phyB-mediated hypocotyl growth despite the fact that it has to cover many time and length scales.

To further mechanistic explanations, it seems feasible to extend the model to e.g. dimers, analysis of rapid regulation of gene expression, posttranscriptional regulation of PIF3 (PIF4, PIF5) levels and levels of phyB itself, which hopefully helps to figure out the signaling component(s). It will be interesting to extend the investigation of phyB dynamics to different spectral light compositions, which establish new photochemical equilibriums, and to see whether adaptation of various ecotypes has used alterations in phyB levels to modulate red light sensitivity of hypocotyl growth inhibition. In conclusion, a quantitative understanding of phytochrome B dynamics is indeed possible. In order to explore further aspects of phyB-mediated photomorphogenesis it will be crucial to pursue the combined approach between experiment and theory exploited in this study, linking biochemistry to plant physiology.

## Materials and Methods

### Plant material, growth conditions

*Arabidopsis thaliana* [ecotype Columbia] wild type (Col WT), mutant (*phyB-9*) and transgenic lines expressing the 35S:PHYB:GFP (called phyB-GFP-1 to phyB-GFP-4 in the text) or 35S:PHYB:YFP (for FRAP experiments) transgenes in *phyB-9* background were used. Construction of the transgenes encoding the phyB-GFP or phyB-YFP fusion proteins, respectively, transformation, selection and regeneration of transgenic lines used in this study has already been described [11], [13]. The selected transgenic lines express fusion proteins that contain the full length phyB protein fused to the fluorescent proteins (GFP or YFP, respectively). The phyB-GFP or phyB-YFP fusion proteins are biologically active photoreceptors as their expression resulted in complementing the *phyB-9* mutant that lacks detectable amount of the phyB photoreceptor [14]. For spectroscopy, the transgenic line ABO/A− was used [49]. Seeds were surface sterilized and sown on Petri-dishes (9 cm diameter) containing 4 layers of filter paper (Macherey-Nagel, Germany) and 4.7 ml distilled water. After stratification (three days at 4°C in darkness) uniform germination was induced by a 4 h white light treatment at 22°C. Afterwards seedlings were grown at 22°C in darkness for four days or subjected to various light treatments as described in the text.

### Light treatments and hypocotyl length measurement

Dark grown seedlings were handled under dim-green safelight [50]. For light pulse treatments and fluence rate response curves modified Leitz Prado 500-W universal projectors (Leitz, Wetzlar, Germany) were used. Red light was obtained by using KG65 filters (λ_max_ = 650 nm; Blazers, Liechtenstein) and far-red light was generated by filtering with an RG9 filter (λ_max_ = 775 nm; Schott, Germany) which adjusts 99.9% Pr [51]. Unless otherwise noted, all other red light treatments were done in a red light field (3 or 22 µmol m^−2^s^−1^) of fluorescent tube lamps (Philips TL 40 W/15) filtered with Plexiglas 501/3 (Röhm und Haas, Darmstadt, Germany). Hypocotyl length of four days old seedlings treated with various light programs as described in the text were measured using ImageJ software (http://rsb.info.nih.gov/ij).

### Time resolved hypocotyl growth curves

Seeds were surface sterilized and sown on Petri-dishes containing 1% Agar with 50% MS medium. The Petri-dishes were kept vertical all time during stratification, germination induction (both as described above) and growth period. During growth period the Petri-dishes were placed between a camera (Marlin F1468) equipped with an infrared filter (IR MTD 52, Tokina Co Limited, Japan) positioned in front, and an infrared light source (Blacklight BL 1960, Advanced illumination) positioned behind the dishes (for set-up see also E. P. Spalding, http://phytomorph.wisc.edu/). Hourly taken pictures, controlled by AVT Smart View 1.7.0 software, monitored hypocotyl growth along the agar surface.

Hypocotyl length of single seedlings was measured over six days with the software ImageJ (http://rsb.info.nih.gov/ij). For growth curves in red light the experimental setup was placed below an intensity controllable LED-panel containing red light LEDs (custom build equipment by the workshop of the Institute of Biology II, University of Freiburg; Red Kingbright, Peak 660 nm, half-width 20 nm, Reichelt Elektronik, Germany). The growth of different seedlings did not start exactly in the same hour after germination induction. To create mean values of the hypocotyl length of 13 seedlings over six days, we first defined a certain hypocotyl length as growth start point, namely 0.5 mm. We estimated the mean hypocotyl growth initiation time point (t*_growth_* = 2111 min) and allocated the new time scale to each seedling. Using the new timescale we achieved an overlay of the growth curves of single seedlings and calculated mean values and standard errors of the hourly measured hypocotyl lengths.

### *In vivo* spectroscopy

Dark reversion of transgenic phyB was measured in complete seedlings in a dual-wavelength ratio spectrophotometer (Ratiospect) [52], [53]. For dark reversion measurement seedlings (ABO/A−) were irradiated with a saturating red light pulse (5 min, 22 µmol m^−2^s^−1^) to create maximal Pfr levels. Subsequently, seedlings were either directly analyzed in the Ratiospect or transferred to darkness for measurement of Pfr levels in prolonged dark incubation. Total amount of phytochrome (Ptot) was measured by six times alternate irradiation with far-red and red light, respectively, followed by absorption measurements that gave the relative amount of photo-convertible phytochrome Δ(ΔA) of the sample. The absorption difference in the sample between the baseline at the beginning of spectroscopy and the first far-red light treatment in the Ratiospect gave the Pfr level of the sample relative to Ptot. For each measurement around 300 seedlings were used and fresh weight was determined directly before the measurement to normalize Δ(ΔA) between the samples. Each time point was measured at least twice. Error bars indicate standard error.

### Fluorescence microscopy

For epifluorescence microscopy, seedlings were transferred to glass slides under green safelight and analyzed with an Axioskop microscope (Zeiss, Oberkochem, Germany). Excitation of phyB-GFP was performed with standard GFP filter sets (AHF Analysetechnik, Tübingen, Germany). Each experiment, light treatments and dark controls, were repeated several times. Cells were documented by photography with a digital Axiocam camera system (Zeiss) during the first 20 seconds of microscopic analysis.

### FRAP experiments

To perform FRAP (fluorescence recovery after photobleaching) experiments seedlings on glass slides were analyzed in a confocal laser scanning microscope equipped with the META detector (LSM 510; Zeiss, Göttingen, Germany). YFP fluorescence was excited by using the 514 nm line of the argon/krypton laser and a 458/514 nm beamsplitter. YFP fluorescence (527–580 nm) was detected using the META detector. After generating overview images of single cells, sub-nuclear areas ( ∼1µm^2^) were photobleached with 100% 514 nm laser power and 25–50 iterations. Subsequently, a time-series of confocal image acquisitions was conducted until the bleached signal reached a stable plateau (in general for 4–6 min). Per time point a z-stack of five images was recorded. Experiments selected for further analysis had to fulfill the following criteria: i) frequent movement of the analyzed foci over time did not exceed the X, Y and Z-limits of the recorded image stacks, and ii) frequent movements of the intranuclear phyB speckles over time did not lead to overlap with other speckles. Prior analysis, the maximum projection algorithm of the LSM software (Zeiss, Göttingen, Germany) was applied to the z-stack data to exclude errors derived by movements of the intranuclear structures in z-direction.

To analyze the FRAP experiments, intensity values of three regions of interest (ROI) were calculated over time, Figure 2D: i) the intensity of the speckle which was actually bleached, ii) the intensity of a speckle of phyB in the same nucleus which was not bleached during the experiment, and iii) the background intensity in an area without fluorescent structures. The raw intensity of the bleached speckle was normalized involving acquisition bleaching correction, i.e., it was corrected by the background intensity. Furthermore, the recovery curve was normalized to the steady state level after bleaching. The recovery curves of five data sets were averaged, such that an average recovery curve with standard errors was used for parameter estimation, see File S1.

### Protein extraction, protein gel blotting and Immunodetection

For phyB accumulation assay seedlings aged 12 h up to four days were used for protein extraction. All other protein extracts were done with four-day-old seedlings. Protein extraction and protein gel blotting was accomplished as described by Bauer et al. [11], except that 1∶100 times plant proteinase inhibitor cocktail (Sigma) was added to the extraction buffer. Immunodetection of phyB was performed using the monoclonal phyB antiserum (B6-B3) as primary antibody. Production and identification of the phyB specific mAb B6-B3 is described by Hirschfeld and colleagues [54]. As secondary antibody either alkaline phosphatase-coupled (BioRad) or horseradish peroxidase-coupled (Vector Labs) anti mouse antiserum was used. Development of the blot was done with ECL-Plus detection System (GE Healthcare) for the light induced phyB degradation assay. Other blots were developed with the Phototrope Star Detection Kit (New England BioLabs). For red light induced phyB degradation experiments, seedlings were irradiated with 1 h, 6 h, 12 h or 24 h red light of 3 µmol m^−2^s^−1^ and harvested together with a non-irradiated control at the end of irradiation period at the age of four days. Signal intensity of dark grown seedlings was set 100%, i.e., 1. Immunoblotting was repeated several times with new series of protein extracts.

### Mathematical modeling and parameter estimation

The phytochrome B dynamics is described on the level of ordinary differential equations using mass action kinetics. We rescaled the concentrations of the systems with the saturated phytochrome level of Col WT in darkness. All simulations were performed with the MATLAB software from MathWorks, Inc. For the integration of the ODE system we used the ode15s function, which was designed to solve stiff differential equations.

To estimate the values of the biological parameters, we chose the following strategies: We determined the degradation rate of Pr, k_dr_, by the transient of the phytochrome accumulation data in dark-grown seedlings of the phyB-GFP-1 line, where the fusion protein is expressed under control of the 35S promoter, see File S1. To keep the model simple, we only considered the overall synthesis, comprising the processes of transcription, translation, and protein association with the chromophore to get the active photoreceptor. Subsequently, the relative steady state levels of phyB-GFP in phyB-GFP-1 and phyB in Col WT were determined by immunoblot analysis, revealing a decreased phytochrome level mediated by the endogenous phyB promoter in wild-type plants. We rescaled the concentrations of the system with the phytochrome dark growth level of Col WT, P_0_^Col^, i.e., the phytochrome level of Col WT is unity and altered levels can be described by P_0_^Mutant^ = zP_0_^Col^, where z is a positive real number and denotes the over-expression strength. We simultaneously estimated the remaining parameters k_3_, k_4_, k_r_, k_dfr_, and the parameters to describe hypocotyl growth in darkness and in light, α_0_β^−1^,γ,L_0_,K′ within a biologically reasonable range by nonlinear least-square fitting on the results of the following independent time-resolved experiments with phyB-GFP/YFP: FRAP experiments (Figure 4A, an appropriate model can be found in File S1), dark reversion kinetics (Figure 4B), red light induced Pfr-degradation (Figure 4C), hypocotyl growth in darkness (Figure 4D), and a light intensity dependent fluence rate response curve under red light irradiation with Col WT (Figure 4E). If necessary, we simulated the pretreatments and the experimental set-ups and used the read-outs under consideration. Further, we assumed that the kinetic parameters of the transgenic over-expressing line phyB-GFP-1 and the Col WT are the same, except for the photoreceptor abundance P_0_.

None of these time-resolved experiments can be directly attributed to the Pr^ns^ speckle depletion with reaction rate k_5_ and the nuclear import rate k_in_. A comprehensive model for the experiment describing the mean number of speckles per nucleus as presented in File S1 would require a detailed knowledge of the coagulation dynamics of phytochrome molecules, which goes far beyond the goal of the present work and will be discussed elsewhere. Due to experimental insufficiencies, it is not possible at the moment to measure the nuclear import rate experimentally. In addition, the sensitivity analysis also revealed that the rate constants k_5_ and k_in_ do not have any influence on the hypocotyl length (Figure 5A). Therefore, we fixed k_5_ and k_in_ in the simulations. The fraction α_0_β^−1^ determines the final hypocotyl length, such that we estimated the corresponding value α_0_ for a fixed β. The mean hypocotyl length L for dark grown seedlings on Petri-dishes containing agar with MS medium (L_A_) to produce time-resolved stem growth differed from the mean hypocotyl length for dark grown seedlings on Petri-dishes containing four layers of filter paper and distilled water (L_P_) to produce fluence rate response curves. Mean hypocotyl length for dark-grown seedlings on agar was L_A_ = 13.29 mm, whereas mean hypocotyl length for dark-grown seedlings on filter paper was L_P_ = 7.58 mm. We interpreted this difference by assuming that the growth limitation on filter paper is stronger than on agar. To overcome this limitation difference and to keep the model as simple as possible, we assumed that β differs by the factor L_P_/L_A_ = 0.69 for the corresponding experimental set-up. In cases of data normalization, the standard errors were re-calculated using Gaussian error propagation.

For each of the five experiments y_j_ = y_j_(t_i_,p), we set up the following merit functions:
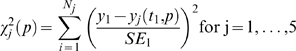
(M1)with y_i_ being the data point at time t_i_ with standard error SE_i_ and y_j_(t_i_,p) being the model value at time point t_i_ with parameter values p. Using the lsqnonlin function from MATLAB, we optimized the overall merit function 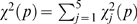 for the different submodels. The corresponding χ^2^-values are summarized in File S1. The 95%-confidence intervals of the estimated parameters were calculated according to Müller et al. [55]. A detailed description of the mathematical modeling of the protein dynamics, the submodels, and the hypocotyl growth model is found in File S1.

## Supporting Information

File S1 Mathematical modeling and additional, experimental information.
(0.46 MB PDF)

Table S1 Phytochrome levels and their corresponding hypocotyl lengths. Relative amount of phytochrome and the corresponding relative hypocotyl length for different over-expressing lines, relative to Col WT in saturating red light conditions. Khanna and colleagues [28] investigated *pif5-2* lines for four days in 15 µmol m^−2^s^−1^; Leivar et al. [29] focussed on *pif7*-set1, *pif3*-set1, *pif3*-set2, *pif4*-set2, *pif3pif7*-set1, *pif4pif7*-set1, and *pif3pif4*-set2 lines in four days old seedlings irradiated with 0.9 µmol m^−2^s^−1^; Al-Sady and colleagues [46] investigated HA:mAPB and *pif3* lines for four days in 9 µmol m^−2^s^−1^. The photoreceptor abundance Col WT and phyB- GFP1 to 4 was determined according to Figure S1B. (−) indicates that no standard error (SE) was determined. The corresponding hypocotyl lengths were measured after four days in 15 µmol m^−2^s^−1^.
(0.03 MB PDF)

Figure S1 Expression levels of phyB. (A) Immunoblot analysis of light induced phyB degradation was performed with four days old seedlings continuous red (cR) light irradiation (3 µmol m^−2^s^−1^). One exemplary blot is shown, quantification of several immunoblots is shown in Figure 4C. (B) For correlation analysis of hypocotyl growth inhibition and phyB level, different levels of Col WT and transgenic lines expressing the 35S:PHYB:GFP transgene (called phyB-GFP-1 to 4) lines were verified by immunoblot data. Coomassie stained SDS-PAGE gel in the lower panel shows equally loaded control.
(0.88 MB TIF)
