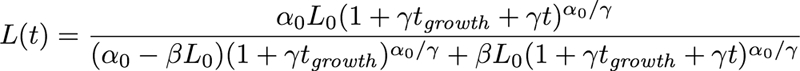# Correction: An Integrative Model for Phytochrome B Mediated Photomorphogenesis: From Protein Dynamics to Physiology

**DOI:** 10.1371/annotation/4563eaf4-e45b-4d9e-ab06-5f1794bf11e3

**Published:** 2010-06-28

**Authors:** Julia Rausenberger, Andrea Hussong, Stefan Kircher, Daniel Kirchenbauer, Jens Timmer, Ferenc Nagy, Eberhard Schäfer, Christian Fleck

Equation 3 contains errors. Please view the correct equation here: