# Supplementary material for: An Integrative Model for Phytochrome B Mediated Photomorphogenesis: From Protein Dynamics to Physiology
Source: PLoS One. 2010 May 19;5(5):e10721. doi: 10.1371/journal.pone.0010721 (PMC2873432; doi:10.1371/journal.pone.0010721)
Supplement: File S1 — Mathematical modeling and additional, experimental information. (0.46 MB PDF) [file pone.0010721.s001.pdf]

Supplementary Information

"An integrative model for phytochrome B mediated photomorphogenesis: from protein dynamics to physiology."

Contents

I. Mathematical Modeling 1

A. Submodels of phytochrome dynamics 1

B. Phytochrome synthesis and degradation 5

C. Determination of kinetic rates from FRAP experiments 6

D. Time-resolved hypocotyl growth 7

II. Experimental supporting information 11

A. Phytochrome synthesis and degradation 11

B. Time-resolved hypocotyl growth 12

C. Counting experiments on fluorescent nuclear bodies 13

References 14

I. MATHEMATICAL MODELING

A. Submodels of phytochrome dynamics

An experimental fluence rate response curve always exhibits the same characteristic features: the hypocotyl growth inhibition seems to be insensitive for rather low and high light intensities. However, for intermediate light intensities, the hypocotyl growth shows a rather sensitive inhibition profile. Furthermore, over-expression lines exhibit rather strong hypocotyl growth inhibition in response to high fluences (Beggs et al., 1980; Wagner and Quail, 1995). Therefore, an appropriate theoretical description of light growth should mimic the characteristic fluence rate response behavior as well as the strong inhibition of over-expression lines for high fluences.

We investigated two submodels of the phytochrome dynamics according to their fluence rate response behavior. The simplest model comprises synthesis, degradation (k_{dr}) and the photochemistry with light intensity (N_λ) dependent transition rates $k_1 = N_\lambda \sigma_r$ and $k_2 = N_\lambda \sigma_{fr}$ of cytosolic phytochrome, cf. Fig. 6A main text with the corresponding rescaled ODEs

$$\begin{aligned} \dot{Pr}^c &= k_{dr} - (k_1 + k_{dr})Pr^c + k_2 Pfr^c \\ \dot{Pfr}^c &= k_1 Pr^c - (k_2 + k_{dfr})Pfr^c. \end{aligned} \tag{S1}$$

To set up a fluence rate responses curve, one has to calculate the amount of Pfr for different values of the light intensity N_λ. The Pfr steady state level, as a function of N_λ, is given by

$$\bar{P}fr(N_\lambda) = \frac{k_{dr}k_1}{k_{dr}(k_2 + k_{dfr}) + k_1 k_{dfr}} = \frac{k_{dr}\sigma_r}{k_{dr}k_{dfr} + N_\lambda(k_{dr}\sigma_{fr} + k_{dfr}\sigma_r)}$$

where $\sigma_{r/fr}$ represent the photoconversion cross-sections (Lagarias et al., 1987). For large light intensities, i.e., for $N_\lambda \to \infty$, the Pfr steady state level saturates to

$$\bar{P}fr(N_\lambda \to \infty) = \frac{k_{dr}\sigma_r}{k_{dr}\sigma_{fr} + k_{dfr}\sigma_r}. \tag{S2}$$

If we consider the simple photochemistry-model, we can fit the following experimental data simultaneously: red light induced Pfr-degradation, fluence rate response curve for Columbia wild type (Col WT), and time-resolved hypocotyl growth in darkness. Then, we predict the fluence rate response curve for the over-expressor phyB-GFP-1 (main text Fig 6C, red dashed line, squares). It can be deduced from the fluence rate response curve that the range of the hypocotyl inhibition for increasing light intensities of the experimental data and the fit and prediction does not match. For very small light intensities, the model of Eqn. (S1) already shows a hypocotyl growth inhibition.

The simplest extension of (S1) is to consider the inactivation step of Pfr^c via dark reversion at rate constant k_r, main text Fig. 6B. The rescaled equations become

$$\begin{aligned} \dot{Pr}^c &= k_{dr} - (k_1 + k_{dr})Pr^c + (k_2 + k_r)Pfr^c \\ \dot{Pfr}^c &= k_1 Pr^c - (k_2 + k_{dfr} + k_r)Pfr^c. \end{aligned} \tag{S3}$$

Again, we calculate the amount of Pfr for different values of the light intensity N_λ. The Pfr

steady state level, as a function of N_λ, is given by

$$\begin{aligned}\bar{P}fr(N_\lambda) &= \frac{k_{dr}k_1}{k_{dr}(k_2 + k_{dfr} + k_r) + k_1 k_{dfr}} \\ &= \frac{k_{dr}\sigma_r}{k_{dr}(k_{dfr} + k_r) + N_\lambda(k_{dr}\sigma_{fr} + k_{dfr}\sigma_r)}.\end{aligned}$$

For $N_\lambda \to \infty$, the *Pfr* steady state level saturates to the same level as Eqn. (S2), but for small and intermediate light intensities, the impact of the dark reversion rate k_r cannot be neglected. If dark reversion is taken into account, we also have to consider the data in the multi-experiment fit, such that we fit the following experiments simultaneously: red light induced *Pfr*-degradation, dark reversion, fluence rate response curve for Col WT, and time-resolved hypocotyl growth in darkness. Again, the prediction is the fluence rate response curve for the over-expressor phyB-GFP-1 (main text Fig. 6C, red dashed line, circles). The fitted and predicted fluence rate response curves for Col WT and phyB-GFP-1, respectively, mimic the sensitivity range of the experimental data much better, especially showing little inhibition for very low fluences.

With the full protein dynamics of Eqns. (1) of the main text, we estimated the parameter combinations by simultaneous nonlinear least-square fitting to the experimental data for all possible $Pfr(t)$ pools and their combinations to represent the signaling player $u(t)$, i.e., assuming the single pools Pfr^c, Pfr^n, Pfr^{ns}, or the sum of the pools, $Pfr^n + Pfr^{ns}$, $Pfr^c + Pfr^n + Pfr^{ns}$ triggering the physiological response. Since all submodels have the same amount of parameters and number of data points, the standard methods for models selection, the Akaike Information Criterion (AIC, (Akaike, 1974)) or the Baysian Information Criterion (BIC, (Schwarz, 1978)), reduce to the classical χ^2-values to give the 'goodness-of-fit', which are summarized in Table S1. Based on this we exclude the cytosolic Pfr^c pool to be the signaling player. The χ^2-values of all other submodels, assuming nuclear pools to be the signaling player, are rather similar, such that we cannot discriminate between these submodels on the basis of the given experiments. This similarity can also be understood on the basis of the mechanistic pool model: Fig. S1 shows the *Pfr*-pool levels, relative to P_{tot}, for different fluences. It can be deduced by the simulations that the pools at different fluences are separated by a scaling factor, such that for the multi-experiment fit the parameters of the hypocotyl growth model can be adapted accordingly. The scaling factor for all fluences can be roughly determined by the kinetic parameters k_3 and k_4, since all other

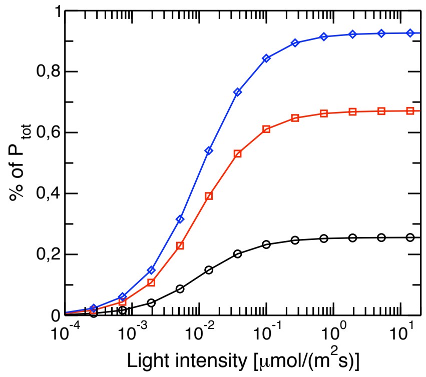

FIG. S1 Simulated Pfr-levels, relative to P_{tot}, for different fluence rates. A scaling factor links the different pools of Pfr^n (black circle), Pfr^{ns} (red square) and $Pfr^n + Pfr^{ns}$ (blue diamond).

process are slow compared to the nuclear body (NB) formation and dissociation. Therefore, the ratio Pfr^n/Pfr^{ns} is given by k_4/k_3, $Pfr^n/(Pfr^n + Pfr^{ns})$ is given by $k_4/(k_3 + k_4)$, and $Pfr^{ns}/(Pfr^n + Pfr^{ns})$ is given by $k_3/(k_3 + k_4)$. Therefore, the multi-experiment fit for different submodels yields similar χ^2-values.

TABLE S1 Model selection using Bayesian Information Criterion (BIC), reduce to the standard χ^2-value, because all submodels have the same amount of parameters and number of data points, which is given by N = 111.

Signaling pool	χ^2
Pfr^c	435.82
Pfr^n	254.21
Pfr^{ns}	254.25
$Pfr^c + Pfr^n + Pfr^{ns}$	254.25
$Pfr^n + Pfr^{ns}$	254.24

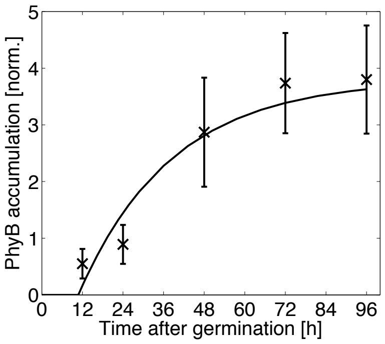

FIG. S2 Synthesis and degradation of phyB-GFP-1. The phyB accumulation (cross) was measured via immunoblot analysis (see Section II.A). The errorbars indicate the standard error of three biological replicates. The solid line represents the fit of Eqn. (S4) to the experimental data.

B. Phytochrome synthesis and degradation

Phytochrome is synthesized in its inactive, cytosolic Pr^c form. The overall synthesis unifies the processes of transcription, translation, and protein association to the chromophore forming the active photoreceptor. To determine the Pr degradation rate k_{dr} on the basis of the transient accumulation dynamics, the level of the total amount of phytochrome was measured via immunoblot analysis, see Section II.A. The phytochrome level can be measured for the first time around 12h after germination induction. Therefore, the determination of the kinetic rates has to be adjusted using the experimental initial conditions $y_0 = x(t_0)$ with t_0 =12 h. The corresponding rescaled ordinary differential equation for phytochrome accumulation for Pr^c in darkness reads

$$\dot{Pr}^c(t) = \Theta(t - t_{syn})zk_{dr} - k_{dr}Pr^c(t) \tag{S4}$$

where $\Theta(t)$ represents a step function, $\Theta(t - t_{syn}) = 1$ for $t > t_{syn}$ and $\Theta(t - t_{syn}) = 0$ otherwise. The estimated time point after germination induction when the measurable synthesis of phytochrome starts is given by $t_{syn} = 648$ min. The photoreceptor abundance of Col WT, P_0^{Col}, is normalized to unity such that the photoreceptor abundance of over-expression lines can be written as $P_0^{Mutant} = zP_0^{Col}$, where $z \in \mathbb{R}^+$ represents the over-expression strength. Fig. S2 represents the experimental data and the fit for total amount of phytochrome in darkness for the over-expression line phyB-GFP-1.

C. Determination of kinetic rates from FRAP experiments

The simultaneous record of a non-bleached reference nuclear body revealed that after bleaching a loss of fluorescence of the non-bleached NB was also observed (main text Fig. 2D, red line). Therefore, for a determination of the recovery curve of the bleached NB, the fluorescence of all remaining NBs and the diffuse fluorescence in the nucleus has to be taken into account.

We consider the following model, where C denotes the nuclear body which is bleached during the FRAP experiment, and R describes the remaining non-bleached NBs. We assume that we have A NBs of approximately the same size. The mean number of NBs, A, can be determined by counting the NBs after continuous red light irradiation. The diffuse fluorescence in the nucleus can be expressed in terms of the total fluorescence, N_{tot}, and the NBs, i.e. $N_{dif} = N_{tot} - C - R$. The following system of ODEs can be derived that describes the association and dissociation dynamics of all NBs

$$\begin{aligned} \dot{R} &= k_3(A-1)N_{tot} - (k_3(A-1)+k_4)R - k_3(A-1)C \\ \dot{C} &= k_3 N_{tot} - (k_3+k_4)C - k_3 R. \end{aligned} \qquad \text{(S5)}$$

The steady state of C is given by $C = k_3 N_{tot}/(Ak_3 + k_4)$. Bleaching of the nuclear body C leads to a loss of the total fluorescence and we denote by θ the fraction of the fluorescence which is still present after the bleaching. If N'_{tot} describes the total fluorescence before bleaching, the total fluorescence after bleaching, N_{tot}, is given by

$$N_{tot} = N'_{tot}\left(1 - \frac{(1-\theta)k_3}{Ak_3+k_4}\right). \qquad \text{(S6)}$$

For the determination of the recovery curve we are interested in the initial conditions directly after bleaching. Normalization with the steady state of C after bleaching yields the following initial conditions

$$C(0) = \frac{\theta(Ak_3+k_4)}{(A-1+\theta)k_3+k_4} \qquad \text{and} \qquad R(0) = \frac{(A-1)(Ak_3+k_4)}{(A-1+\theta)k_3+k_4}$$

and the normalized ODE system reads

$$\begin{aligned} \dot{R} &= (A-1)(Ak_3+k_4) - (k_3(A-1)+k_4)R - k_3(A-1)C \\ \dot{C} &= Ak_3 + k_4 - (k_3+k_4)C - k_3 R. \end{aligned} \qquad \text{(S7)}$$

System (S7) is an inhomogeneous, linear system of ODEs whose solution can be calculated using the method of variation of constants and are given by

$$R(t) = c_1 e^{-(Ak_3+k_4)t} + c_2 e^{-k_4 t} + (A-1)(1 - e^{-(Ak_3+k_4)t}) \tag{S8}$$

$$C(t) = c_1 e^{-(Ak_3+k_4)t} - c_2 e^{-k_4 t} + 1 - e^{-(Ak_3+k_4)t} \tag{S9}$$

with

$$c_1 = \frac{(A-1)(C(0)+R(0))}{A} = \frac{(A-1+\theta)(Ak_3+k_4)}{A[(A-1+\theta)k_3+k_4]},$$

$$c_2 = \frac{R(0)-(A-1)C(0)}{A} = \frac{(A-1)(1-\theta)(Ak_3+k_4)}{A[(A-1+\theta)k_3+k_4]}.$$

The recovery curve of the bleached NB under consideration can be described by Eqn. (S9), but the question arises if the parameters k_3 and k_4 are identifiable. If we find more than one NB in the nucleus, which is always the case for the phytochrome system, the dynamics of the recovery curve becomes insensitive to changes of the association rate k_3, i.e., k_3 cannot be determined by Eqn. (S9). Fig. S3A shows the recovery curve $C(t)$ varying k_3 from 0.001 to 10, i.e., over four orders of magnitude, under the assumption of more than one NB. The recovery curves for different k_3 do not differ significantly. The dissociation rate k_4, however, is identifiable and therefore can be determined by the recovery curve described by Eqn. (S9) as it can be seen in Fig. S3B. Varying k_4 again over four orders of magnitude, the recovery curves look very different. The non-identifiability of k_3 can be explained in rather rational terms based on Eqn. (S9): for $k_3 \gg k_4$, some large complexes are present and the transient recovery dynamics of the bleached complex only depends on the release of fluorescent molecules from the remaining ones; for $k_3 \ll k_4$, a lot of small complexes are present, such that the exchange of only some fluorescent molecules suffices to reach the final steady state level given by $k_3/(k_3 + k_4)$. Therefore, the recovery of the bleached complex almost exclusively depends on the release of fluorescent molecules of the remaining complexes rather than on the association of diffusive fluorescent molecules to the bleached complex.

D. Time-resolved hypocotyl growth

In 1838, Verhulst introduced the logistic growth equation to describe growth in the presence of environmental limitations. The rate of reproduction is proportional to the existing

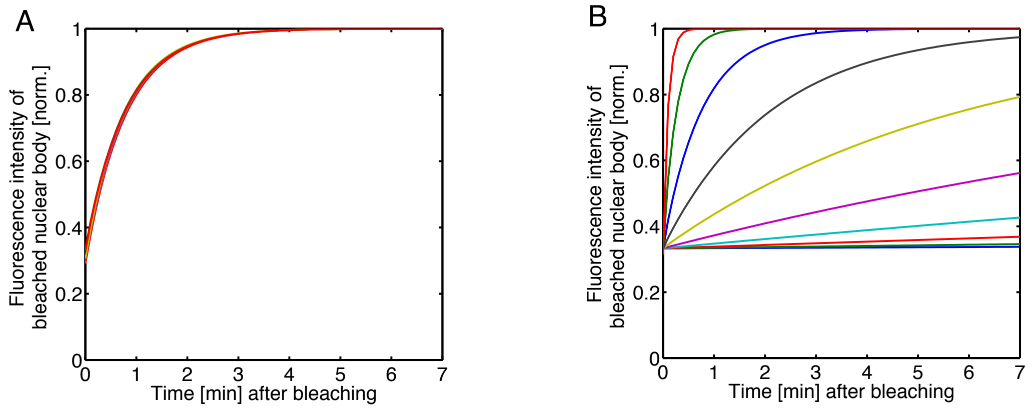

FIG. S3 Recovery curves according to Eqn. (S9), assuming $\theta = 0.29, A = 5.5$ and (A) varying $k_3 \in [0.001, 10]$. The recovery curve does not depend on k_3. (B) The effect of varying $k_4 \in [0.001, 10]$ is reflected by the recovery curve.

population and the amount of available resources. Therefore, a modified logistic growth function for the description of hypocotyl growth is given by

$$\dot{L}(t) = (\alpha - \beta L(t))L(t). \tag{S10}$$

For small times and small initial hypocotyl length $(L_0(t_0) < \alpha/\beta)$, the hypocotyl grows exponentially with growth rate $\alpha - \beta L_0$. For larger times, growth is inhibited by environmental factors, such that the inhibition factor $\beta L(t)$ dominates and the hypocotyl length reaches its final steady state level, given by $L(t \to \infty) = \alpha/\beta$. The solution of (S10) reads

$$L(t) = \frac{\alpha L_0}{\beta L_0 + e^{-\alpha(t-t_0)}(\alpha - \beta L_0)}. \tag{S11}$$

If $L_0(t_0) < \alpha/\beta$, the solution (S11) describes an increasing, sigmoidal, i.e., S-shaped, and symmetric curve with inflection point $t_I = \alpha/(2\beta)$. The inflection point of the growth curve also describes the maximum of the growth velocity function.

However, experimental investigations in the late 19th and early 20th centuries of growth patterns revealed that in general, a growth curve is not symmetric with respect to the inflection point (for a detailed overview see Backman (1931)). Julius von Sachs described in 1874 the growth pattern as a “large period of growth”, being characterized by an initially increasing growth velocity that reaches a maximum and is followed by a finite decrease of the growth velocity. Therefore, in 1943 Backman introduced the so-called “organic time”, meaning that the growth rate decreases with increasing age of the organism. The growth cycle again can

be described by an S-shaped function, which is – in contrast to Verhulst's logistic growth function – not symmetric with respect to the inflection point, cf. Fig. S4A. Therefore, the corresponding velocity function is an asymmetric bell-shaped function, reflecting that the period of increasing velocity is of greater amplitude than the period of decreasing velocity, cf. Fig. S4B. In 1965, Hock and Mohr used a modified version of the "organic time", but estimated some model parameters for dark and light growth separately, since the effect of phyotchrome on hypocotyl growth was completely unknown. Using the ideas of Backman, and Hock and Mohr, we describe the growth function for dark grown seedlings by

$$\dot{L}(t) = \frac{\Theta(t - t_{growth})}{1 + \gamma t}(\alpha_0 - \beta L(t))L(t). \tag{S12}$$

Before deriving the growth function for light grown seedlings, we give some characteristic features of the introduced factor γ, and set $t_{growth} = 0$. For $\gamma = 0$, we obtain the logistic growth function described by (S10). An increase of γ for $\gamma > 0$ makes the growth curve less steep (cf. Fig. S4A), which seems to be a better description of experimental growth patterns (Backman, 1943). If we ignore environmental limitations, i.e., if $\beta = 0$, the solution of (S12) is given by $L(t) = L_0(1 + \gamma t)^{\alpha_0/\gamma}$. Therefore the fraction α_0/γ determines the type of growth for small times, i.e., for $\alpha_0 > \gamma$ we obtain exponential growth, for $\alpha_0 = \gamma$ we obtain linear growth, and for $\alpha_0 < \gamma$ the growth is sublinear, cf. Fig. S4C.

The intuitive and most simple ansatz would be to modify the growth rate α_0 to $\alpha(t) = \alpha_0/(1 + Ku(t))$, where $u(t)$ represents the rescaled signaling phytochrome component as described in the main text. All experiments for the multi-experiment fit can be described rather well with the above growth rate. However, the prediction of the high fluences of the over-expression line phyB-GFP-1 lies far above the experimental data (Fig. S5, red dashed line). Therefore, we modify α_0 to $\alpha(t) = \alpha_0/(1 + K^2u^2(t))$. Since all rescaled phytochrome components depend linearly on the over-expression strength z of the phytochrome abundance P_0^{Col}, the modified growth rate $\alpha(t)$ is a decreasing function for an increasing amount of phytochrome, being rather insensitive to small amounts of phytochromes, $z \ll 1$. For intermediate over-expression of the photoreceptor abundance, the growth rate diminishes and converges to zero for strong over-expression of the photoreceptor abundance. In the absence of active phytochrome $\alpha(t)$ reduces to the originial α_0, cf. Eqn. (S12). Therefore,

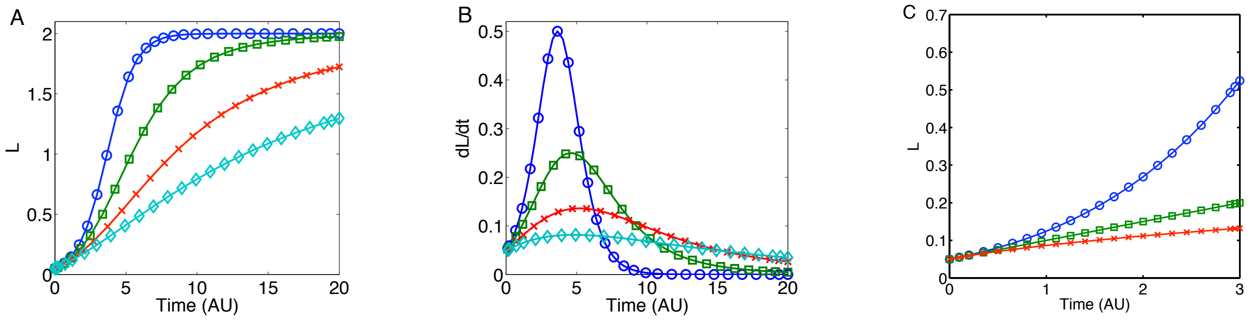

FIG. S4 (A) Growth curves and (B) growth velocity functions for different values of γ ($\gamma = 0$, blue circle; $\gamma = 0.2$, green square; $\gamma = 0.4$, red plus; $\gamma = 0.6$, cyan diamond). The greater γ, the more asymmetric is the growth velocity function and the later reaches the growth curve the steady state. (C) The fraction α_0/γ determines the shape of the growth function for small times t. For $\alpha_0 > \gamma$ (blue circle), the initial growth is rather exponential; for $\alpha_0 = \gamma$ (green square), the growth is linear; for $\alpha_0 < \gamma$ (red plus), the initial growth is sublinear.

the hypocotyl growth of light grown seedlings can be described by the following function

$$\dot{L}(t) = \frac{\Theta(t - t_{growth})}{1 + \gamma t} \left(\frac{\alpha_0}{1 + K^2 u^2(t)} - \beta L(t) \right) L(t) = \mu(t)L(t) - \nu(t)L(t)^2 \tag{S13}$$

with

$$\mu(t) = \frac{\Theta(t - t_{growth})}{1 + \gamma t} \frac{\alpha_0}{1 + K^2 u^2(t)} \qquad \text{and} \qquad \nu(t) = \frac{\Theta(t - t_{growth})\beta}{1 + \gamma t}.$$

The hypocotyl grows, if $\alpha_0/\beta - 1 > K^2 u^2(t)$, such that the general solution of (S13) is given by

$$L(t) = \frac{L_0 e^{\int_0^t \mu(t')dt'}}{1 + L_0 \int_0^t e^{\int_0^t \mu(t'')dt''} \nu(t')dt'}. \tag{S14}$$

Due to the experimental observation that phytochrome synthesis can be observed approx. 12 h after germination induction (estimated $t_{syn} = 648$ min) whereas the hypocotyl starts to grow approx. 35 h after germination induction ($t_{growth} = 2111$ min), the protein dynamics reaches the steady state very fast in comparison to hypocotyl growth. Therefore, the time-dependent solution of the rescaled signaling component $u(t)$ becomes time-independent with $\bar{u}$ as the steady state level, and the modified growth reads $\alpha' = \alpha_0/(1 + K^2\bar{u}^2)$. The solution for (S13) with $t > t_{growth}$ then reads

$$L(t) = \frac{\alpha' L_0 (1 + \gamma t)^{\alpha'/\gamma}}{\alpha' - \beta L_0 + \beta L_0 (1 + \gamma t)^{\alpha'/\gamma}}. \tag{S15}$$

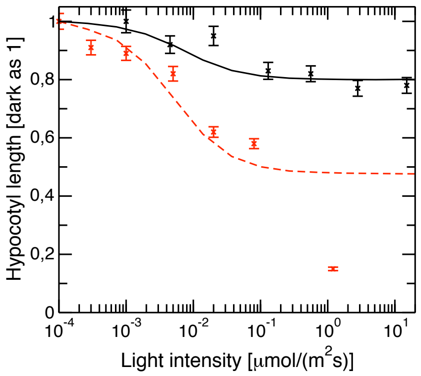

FIG. S5 Prediction of the fluence rate response curve of the phyB-GFP-1 over-expressor (red dashed line) and the corresponding experimental data, when assuming the modified growth rate to be $\alpha(t) = \alpha_0/(1 + Ku(t))$. The fluence rate response curve for Col WT (black solid line) was subject to the multi-experiment fit described in the main text.

The read-out of most of the experiments is an end-point of the hypocotyl. This end-point either describes the steady state level of the hypocotyl (for $t > t_c$) or an intermediate hypocotyl length (for $t < t_c$) for some critical time $t_c = [(\alpha'/(\beta L_0) + 1)^{\gamma/\alpha'} - 1]/\gamma$ and is given by

$$L(t) = \frac{\alpha'}{\beta} = \frac{\alpha_0}{\beta(1 + K^2\bar{u}^2)} \quad \text{for} \quad t \gg t_c \tag{S16}$$

$$L(t) = L_0 e^{\frac{\alpha'}{\gamma}\ln(1+\gamma t)} \quad \text{for} \quad t_{growth} < t < t_c. \tag{S17}$$

II. EXPERIMENTAL SUPPORTING INFORMATION

A. Phytochrome synthesis and degradation

PhyB accumulation was determined with phyB specific monoclonal antibodies in seedlings at the age of 12 h up to four days. In the first days of seedling development growth is achieved by water uptake, thus the water content of the seedlings is increasing. To compensate this difference between the samples we used exactly the same number of seeds per sample, weighed the seeds prior to imbibition and directly before harvesting. Thus we could caluclate the water uptake of each sample. By using always the same volume of extraction buffer and taking the water uptake into account, the dilution factor of each protein extract caused by seedling‘s water uptake was obtained. Afterwards SDS-PAGE gel was loaded

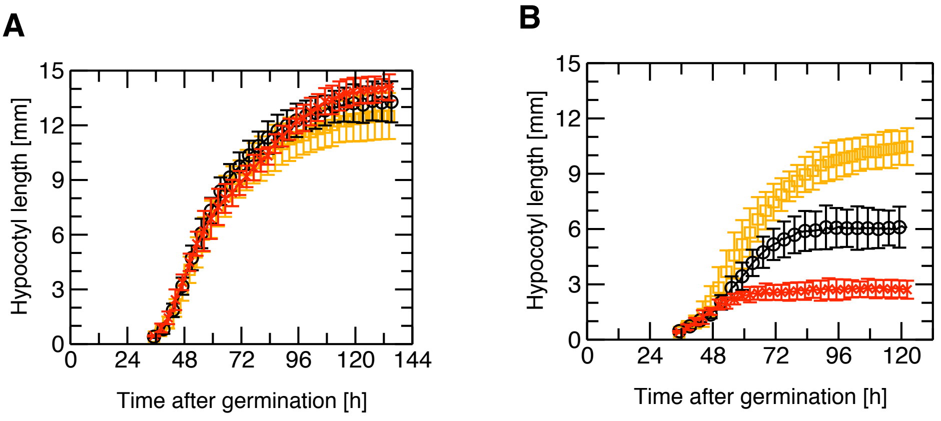

FIG. S6 Time-resolved hypocotyl growth in darkness (A) and in continuous red light of 3.8 μmol/(m^{2}s) (B) for *phyB-9* mutant (orange square), Col WT (black circle) and phyB-GFP-1 (red cross). The experimental set-up is described in the Materials and Methods in the main text.

with 20 μl of the earliest sample (12 h) and 20 μl multiplied with the dilution factor for the following extracts. The first measurable phytochrome signal appeared around 12 to 24 h after germination induction. Up to the third day the phyB amount increased, at the fourth day it reached a slightly lower plateau.

B. Time-resolved hypocotyl growth

In addition to the dark growth curve presented in Fig. 4D in the main text, we measured time-resolved seedling growth in continuous red light of 3.8 μmol/(m^{2}s). Experimental setup for growth curves is explained in Materials and Methods in the main text. Fig. S6A shows the dark growth curves of 3 genotypes, *phyB-9* mutant, Col WT and 35S:phyB:GFP over-expressing line (phyB-GFP-1). As indicated in the main text, the S-shaped dark growth curves of the analyzed genotypes were very similar and did not show diurnal rhythm. Continuous irradiation with 3.8 μmol/(m^{2}s), as expected, resulted in a dramatic difference between the growth of the analyzed genotypes, Fig. S6B. Light grown *phyB-9* mutant seedlings did not show any difference to dark growth. However, the maximal growth of Col WT was strongly reduced and the maximal growth of phyB-GFP-1 was even more reduced. Again, the light grown seedlings did not show rhythmic changes of growth velocity in our experimental conditions.

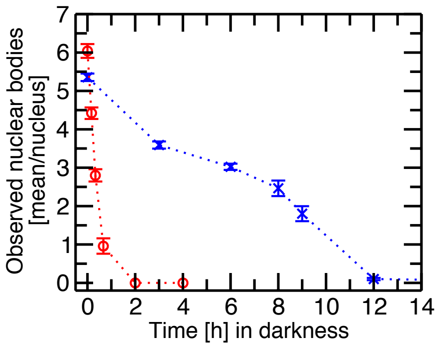

FIG. S7 Using fluorescence microscopy, the Pr^{ns} (red circle) and Pfr^{ns} (blue cross) dissociation was determined over time indirectly by counting the mean amount of nuclear bodies after a NB-inducing treatment that was ended either with (red circle) or without (blue cross) a Pr creating RG9 pulse, followed by darkness.

C. Counting experiments on fluorescent nuclear bodies

Using fluorescence microscopy, we counted the mean number of NBs in the *Arabidopsis* 35S:PHYB:GFP expressing line after a nuclear body inducing light treatment that was ended with or without a Pr creating RG9 pulse, followed by darkness. If the light treatment was ended without an RG9 pulse, the Pfr NBs were visible over at least 9 hours of prolonged darkness, Fig. S7 (blue cross). In contrary, if an RG9 pulse was given at the end of the light treatment, the Pr NBs already dissolved within 1 hour of darkness, Fig. S7 (red circle).
To describe the counting experiments of the NBs after different irradiation scenarios, a comprehensive model for the coagulation dynamics of the phytochrome molecules, and the connection to the pool dynamics with rate constant k_5 is needed. This descriptions goes far beyond the goal of the present manuscript and will be discussed elsewhere.

References

Akaike H (1974) A new look at the statistical model identification. *IEEE Trans Automatic Control* **19**: 716–723

Backman G (1931) Das Wachstumsproblem. *Erg Physiol* **33**: 883–937

Backman G (1943) *Wachstum und organische Zeit.* Leipzig: Barth

Beggs CJ, Holmes MG, Jabben M, Schäfer E (1980) Action spectra for the inhibition of hypocotyl growth by continuous irradiation in light- and dark-grown *Sinapis alba* L. seedlings. *Plant Physiol* **66**: 615–618

Hock B, Mohr H (1965) A quantitative analysis of growth processes in connection with photomorphogenesis in mustard seedlings (*Sinapis alba L.*). *Planta* **65**: 1–16

Lagarias JC, Kelly JM, Cyr KL, Smith WO (1987) Comparative photochemical analysis of highly purified 124-kilodalton oat and rye phytochromes *in vitro. Photochem Photobiol* **46**: 5–13

Schwarz G (1978) Estimating the dimension of a model. *Ann Statist* **6**: 461–464

Verhulst PF (1838) Notice sur la loi que la population poursuit dans son accroissement. *Corresp math et phys* **10**: 113–121

Wagner D, Quail PH (1995) Mutational analysis of phytochrome B identifies a small COOH-terminal-domain region critical for regulatory activity. *Proc Natl Acad Sci U S A* **92**: 8596–8595
