# Supplementary material for: An Integrative Model for Phytochrome B Mediated Photomorphogenesis: From Protein Dynamics to Physiology
Source: PLoS One. 2010 May 19;5(5):e10721. doi: 10.1371/journal.pone.0010721 (PMC2873432; doi:10.1371/journal.pone.0010721)
Supplement: Table S1 — Phytochrome levels and their corresponding hypocotyl lengths. Relative amount of phytochrome and the corresponding relative hypocotyl length for different over-expressing lines, relative to Col WT in saturating red light conditions. Khanna and colleagues [28] investigated pif5-2 lines for four days in 15 µmol m−2s−1; Leivar et al. [29] focussed on pif7-set1, pif3-set1, pif3-set2, pif4-set2, pif3pif7-set1, pif4pif7-set1, and pif3pif4-set2 lines in four days old seedlings irradiated with 0.9 µmol m−2s−1; Al-Sady and colleagues [46] investigated HA:mAPB and pif3 lines for four days in 9 µmol m−2s−1. The photoreceptor abundance Col WT and phyB- GFP1 to 4 was determined according to Figure S1B. (−) indicates that no standard error (SE) was determined. The corresponding hypocotyl lengths were measured after four days in 15 µmol m−2s−1. (0.03 MB PDF) [file pone.0010721.s002.pdf]

TABLE S1 **Phytochrome levels and their corresponding hypocotyl lengths.** Relative amount of phytochrome and the corresponding relative hypocotyl length for different overexpressing lines, relative to Col WT in saturating red light conditions. Khanna and colleagues [28] investigated *pif5-2* lines for four days in 15 μmol/(m^{2}s); Leivar et al. [29] focussed on *pif7*-set1, *pif3*-set1, *pif3*-set2, *pif4*-set2, *pif3pif7*-set1, *pif4pif7*-set1, and *pif3pif4*-set2 lines in four days old seedlings irradiated with 0.9 μmol/(m^{2}s); Al-Sady and colleagues [46] investigated HA:mAPB and *pif3* lines for four days in 9 μmol/(m^{2}s). The photoreceptor abundance Col WT and phyB-GFP1 to 4 was determined according to Figure S1B. (–) indicates that no standard error (SE) was determined. The corresponding hypocotyl lengths were measured after four days in 15 μmol/(m^{2}s).

Description	Photoreceptor abundance mean (SE)	Hypocotyl length mean (SE)
Col WT	1 (–)	1 (0.03)
pif7-set1	1.11 (0.03)	0.76 (0.04)
HA:mAPB	1.468 (0.026)	0.514 (0.023)
pif3	1.516 (0.051)	0.502 (0.024)
pif3-set2	1.50 (0.07)	0.57 (0.03)
pif3-set1	1.59 (0.07)	0.58 (0.03)
pif4-set2	1.61 (0.04)	0.52 (0.03)
pif5-2	1.63 (0.05)	0.69 (0.09)
pif4-set1	1.77 (0.13)	0.5 (0.02)
pif3pif7-set1	2.11 (0.04)	0.48 (0.02)
pif4pif7-set1	2.3 (0.18)	0.47 (0.02)
pif3pif4-set2	3.04 (0.18)	0.38 (0.02)
phyB-GFP-1	3.8 (–)	0.26 (0.03)
phyB-GFP-4	4.7 (–)	0.23 (0.005)
phyB-GFP-2	5.3 (–)	0.19 (0.03)
phyB-GFP-3	5.4 (–)	0.19 (0.01)
