# Supplementary figures and images for: An Integrative Model for Phytochrome B Mediated Photomorphogenesis: From Protein Dynamics to Physiology
Source: PLoS One. 2010 May 19;5(5):e10721. doi: 10.1371/journal.pone.0010721 (PMC2873432; doi:10.1371/journal.pone.0010721)

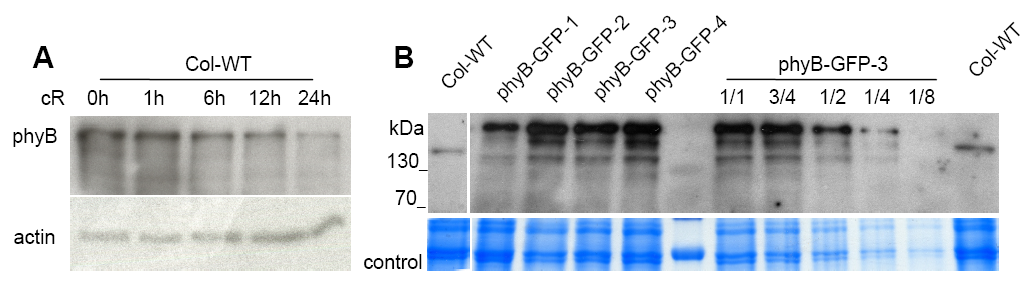

Supplement: Figure S1 — Expression levels of phyB. (A) Immunoblot analysis of light induced phyB degradation was performed with four days old seedlings continuous red (cR) light irradiation (3 µmol m−2s−1). One exemplary blot is shown, quantification of several immunoblots is shown in Figure 4C. (B) For correlation analysis of hypocotyl growth inhibition and phyB level, different levels of Col WT and transgenic lines expressing the 35S:PHYB:GFP transgene (called phyB-GFP-1 to 4) lines were verified by immunoblot data. Coomassie stained SDS-PAGE gel in the lower panel shows equally loaded control. (0.88 MB TIF) [file pone.0010721.s003.tif]
